# Inorganic carbon and pH dependency of photosynthetic rates in *Trichodesmium*

**DOI:** 10.1093/jxb/ery141

**Published:** 2018-04-12

**Authors:** Tobias G Boatman, Niall M Mangan, Tracy Lawson, Richard J Geider

**Affiliations:** 1 School of Biological Sciences, University of Essex, Wivenhoe Park, Colchester, Essex, UK; 2 Department of Engineering Sciences and Applied Mathematics, Northwestern University, Evanston, Illinois, USA

**Keywords:** Carbon acquisition, carbon concentrating mechanism (CCM), CO_2_, Cyanobacteria, gross photosynthesis, net photosynthesis, ocean acidification, *Trichodesmium*

## Abstract

Increasing atmospheric CO_2_ concentrations are leading to increases in dissolved CO_2_ and HCO_3_^–^ concentrations and decreases in pH and CO_3_^2–^ in the world’s oceans. There remain many uncertainties as to the magnitude of biological responses of key organisms to these chemical changes. In this study, we established the relationship between photosynthetic carbon fixation rates and pH, CO_2_, and HCO_3_^–^ concentrations in the diazotroph, *Trichodesmium erythraeum* IMS101. Inorganic ^14^C-assimilation was measured in TRIS-buffered artificial seawater medium where the absolute and relative concentrations of CO_2_, pH, and HCO_3_^–^ were manipulated. First, we varied the total dissolved inorganic carbon concentration (TIC) (<0 to ~5 mM) at constant pH, so that ratios of CO_2_ and HCO_3_^–^ remained relatively constant. Second, we varied pH (~8.54 to 7.52) at constant TIC, so that CO_2_ increased whilst HCO_3_^–^ declined. We found that ^14^C-assimilation could be described by the same function of CO_2_ for both approaches, but it showed different dependencies on HCO_3_^–^ when pH was varied at constant TIC than when TIC was varied at constant pH. A numerical model of the carbon-concentrating mechanism (CCM) of *Trichodesmium* showed that carboxylation rates are modulated by HCO_3_^–^ and pH. The decrease in assimilation of inorganic carbon (Ci) at low CO_2_, when TIC was varied, was due to HCO_3_^–^ uptake limitation of the carboxylation rate. Conversely, when pH was varied, Ci assimilation declined due to a high-pH mediated increase in HCO_3_^–^ and CO_2_ leakage rates, potentially coupled to other processes (uncharacterised within the CCM model) that restrict Ci assimilation rates under high-pH conditions.

## Teaser

Photosynthetic rates in the cyanobacterium *Trichodesmium erythraeum* appear to be a function of CO_2_, although numerical simulations of the carbon-concentrating mechanism show carboxylation to be mediated as a function of pH and HCO_3_^–^.

## Introduction

Over the past 150 years, atmospheric CO_2_ concentrations have increased from pre-industrial levels (i.e. 280 µmol mol^–1^) to a current value of about 400 µmol mol^–1^, and are predicted to increase further to 650 µmol mol^–1^ by mid-century, and to 750–1000 µmol mol^–1^ by the end of this century (Raven *et al.*, 2005). Equilibration of CO_2_ between the atmosphere and the oceans is leading to increases in dissolved CO_2_ and HCO_3_^–^ and to decreases in pH and CO_3_^2–^. This process of ocean acidification is predicted to reduce the pH from average pre-industrial levels of 8.2 to about 7.9 by the end of the century (Zeebe *et al.*, 1999; Zeebe and Wolf-Gladrow, 2001). To date, there are still many uncertainties as to the magnitude of biological responses of key organisms to these chemical changes.

One group of organisms of particular importance are the diazotrophic cyanobacteria (photosynthetic dinitrogen-fixers), notably because of their significant contribution to marine primary productivity by converting N_2_ into NH_4_^+^, thus providing ‘new’ nitrogen to the oceans. The filamentous cyanobacterium *Trichodesmium* is a colony-forming species that fixes nitrogen in an area corresponding to half the Earth’s surface (Davis and McGillicuddy, 2006), and is estimated to account for more than half of the new (combined) nitrogen production in many parts of the oligotrophic tropical and sub-tropical oceans (Capone *et al.*, 2005).

Cyanobacteria, *Trichodesmium* species included, achieve high photosynthetic rates despite (i) the slow diffusion of CO_2_ in water (10^4^ times slower than in air), (ii) a slow chemical equilibrium between HCO_3_^–^ and CO_2_ within the 7–8.5 pH range, and (iii) a low affinity of Rubisco for CO_2_ relative to ambient CO_2_ concentrations. Cyanobacteria employ an intracellular carbon-concentrating mechanism (CCM) (Badger and Price, 2003; Badger *et al.*, 2006; Kranz *et al.*, 2010), where enhanced primary productivity significantly outweighs the metabolic costs of CCM activity (Price *et al.*, 2008). The CCM benefits cyanobacteria by reducing photorespiration (Schwarz *et al.*, 1995; Kaplan and Reinhold, 1999), aiding in the dissipation of excess light energy, and by maintaining an optimal intracellular pH (Badger *et al.*, 1994; Kaplan and Reinhold, 1999). The general consensus is that up-regulation of CCM activity in response to a low-CO_2_ environment involves two components. Firstly, an increase in the transport of inorganic carbon (Ci) from the environment into the cell via a suite of Ci transporters, which could involve using ATP (BCT1 HCO_3_^–^ transporter), NADPH, or reduced ferredoxin (CO_2_ conversion from passive diffusion) or coupling to an electrochemical Na^+^ gradient (SbtA or BicA HCO_3_^–^ transport) to provide the energy for Ci uptake (Badger *et al.*, 2002; Badger and Price, 2003). Secondly, an increased ability to reduce CO_2_ leakage from around the site of carboxylation, achieved via arrangement of the molecular components of the carboxysome structure and a CO_2_-uptake system located on the thylakoid layer, preventing the efflux of leaked CO_2_ to the outer cytosolic layer (Price *et al.*, 2008).

Both ^14^C isotope disequilibrium experiments and simultaneous measurements of CO_2_ and O_2_ exchanges during sequential light–dark transitions indicate that HCO_3_^–^ contributes >90% of the Ci assimilation by *T. erythraeum* IMS101 (Kranz *et al.*, 2009; Eichner *et al.*, 2015). This preference for HCO_3_^–^ is consistent with the evidence that *Trichodesmium* lacks a plasma membrane-bound extracellular carbonic anhydrase (eCA) (Badger *et al.*, 2006; Price *et al.*, 2008). Furthermore, the *T. erythraeum* genome indicates the presence of both a plasma membrane HCO_3_^–^ transporter (BicA) and an intracellular system for conversion of CO_2_ to HCO_3_^–^ (NDH-I_4_) (Price *et al.*, 2008). These two modes of the CCM result in the accumulation of HCO_3_^–^ in the cytosol, which diffuses to the carboxysome. Inorganic carbon uptake by *Trichodesmium* involves the uptake of HCO_3_^–^ by the BicA transporter. This transporter has a half-saturation constant, *K*_m_, of 40–100 µM HCO_3_^–^, which is well below the typical concentration of HCO_3_^–^ in seawater (~2000 µM) (Badger *et al.*, 2006). Following transport into the cell, C-fixation in *Trichodesmium*, like other cyanobacteria species, occurs within carboxysomes where HCO_3_^–^ is converted to CO_2_ via a carbonic anhydrase, followed by fixation of CO_2_ by Rubisco. Carboxysomes provide micro-environments where CO_2_ is elevated to compensate for the low affinity of cyanobacterial Rubiscos for CO_2_ (K_m_CO_2_>150 mM) (Badger and Andrews, 1987). In *Trichodesmium*, CO_2_ that leaks from carboxysomes can be converted to HCO_3_^–^ by the plasma membrane-bound NDH-I_4_ protein, thus reducing the efflux of CO_2_ from the cell, but at a cost of consuming reducing equivalents (NADPH or reduced Fd) (Price *et al.*, 2008). Despite having a mechanism for intracellular recycling of CO_2_, efflux is reported to account for the loss of up to 50% of HCO_3_^–^ uptake in *Trichodesmium* (Kranz *et al.*, 2010; Eichner *et al.*, 2015).

As reviewed in Boatman *et al.* (2017), the majority of previous studies have shown an increase (albeit not all statistically significant) in *T. erythraeum* IMS101 growth under predicted future CO_2_ concentrations (~750–1000 µmol mol^–1^), although the magnitudes of these responses differ between studies (see Supplementary Table S1 at *JXB* online). The increased growth and productivity of *T. erythraeum* IMS101 with increased CO_2_ is probably attributable to a decrease in the energy required for operation of the CCM, allowing more energy (ATP) and reductant (NADPH) to be reallocated to N_2_ fixation, CO_2_ fixation, and biosynthesis (Kranz *et al.*, 2011).

Given the significant contribution of *Trichodesmium* to carbon and nitrogen biogeochemical cycles, and the predicted changes to Ci speciation over the coming decades due to ocean acidification, we performed a systematic study to assess how the kinetics of Ci assimilation of *T. erythraeum* IMS101 were affected by acclimation to varying CO_2_ concentrations. We ensured that the Ci chemistry and all other growth conditions were well defined, with cultures fully acclimated over long time periods to achieve balanced growth. We assessed how the rate of Ci assimilation was related to CO_2_ or HCO_3_^–^ concentrations in experiments where Ci speciation was modulated by varying pH and total dissolved inorganic carbon concentration (TIC). These assays of photosynthetic performance showed that *Trichodesmium* productivity was influenced by high pH when TIC was held at a saturating concentration, indirectly making the rate of Ci assimilation a saturating function of CO_2_ concentration, and that maximum rates of CO_2_ fixation declined and affinity for CO_2_ increased when *Trichodesmium* was acclimated to a low-CO_2_ concentration. We discuss how these responses can be attributed to decreases in the cost of operating a CCM under future CO_2_ conditions.

## Materials and methods

*Trichodesmium erythraeum* IMS101 was semi-continuously cultured to achieve fully acclimated balanced growth at three target CO_2_ concentrations (180, 380, and 720 µmol mol^–1^) under saturating light intensity (400 µmol photons m^–2^ s^–1^), a 12/12 h light/dark (L/D) cycle, and an optimum growth temperature (26 ± 0.7 °C) for ~5 months (~80 generations).

### Experimental set-up

Cultures of *T. erythraeum* IMS101 were grown using YBCII medium (Chen *et al.*, 1996) at 1.5-l volumes in 2-l Pyrex bottles that had been acid-washed and autoclaved prior to culturing. Daily growth rates were quantified from changes in baseline fluorescence (*F*_o_) measured between 09.00 to 10.30 h on dark-adapted cultures (20 min) using a FRRfII FastAct Fluorometer System (Chelsea Technologies Group Ltd, UK). Cultures were kept at the upper section of the exponential growth phase through periodic dilution with new growth media at 3–5 d intervals. They were deemed fully acclimated and in balanced growth when both the slope of the linear regression of ln(*F*_o_) and the ratio of live-cell to acetone-extracted (method detailed below) baseline fluorescence were constant following every dilution with fresh YBCII medium. Illumination was provided side-on by fluorescent tubes (Sylvania Luxline Plus FHQ49/T5/840). Cultures were constantly mixed using magnetic PTFE stirrer bars and aerated with a filtered (0.2-µm pore) air mixture at a rate of ~200 ml s^–1^. The CO_2_ concentration was regulated (±2 µmol mol^–1^) by mass-flow controllers (Bronkhorst, Newmarket, UK) and CO_2_-free air was supplied by an oil-free compressor (Bambi Air, UK) via a soda-lime gas-tight column that was mixed with a 10% CO_2_-in-air mixture from a gas cylinder (BOC Industrial Gases, UK). The CO_2_ concentration in the gas phase was continuously monitored and recorded by an infra-red gas analyser (Li-Cor Li-820, Nebraska USA), calibrated weekly against a standard gas (BOC Industrial Gases).

The Ci chemistry was measured prior to the dilution of each culture with fresh media; pH and TIC were measured directly, while HCO_3_^–^, CO_3_^2–^, and CO_2_ concentrations were calculated using CO2SYS with the same constants as described in Boatman *et al.* (2017) (see Supplementary Information SI).

### Elemental stoichiometry

Samples for elemental composition and CO_2_-response curves were collected at the same time of day between 4 and 6 h into the light period of the L/D cycle. Samples for determination of particulate organic carbon (POC), particulate nitrogen (PN), and particulate phosphorus (PP) were collected together with each CO_2_-response curve, where each sample was a biological replicate culture. Three 100-ml aliquots from each culture were vacuum-filtered onto pre-combusted 25-mm (0.45-µm pore) glass-fibre filters for measurements of POC, PN, and PP. The POC and PN filters were placed in 1.8-ml cryovials (lids off) and dried at 60 °C. The PP filters were rinsed with 2 ml of sodium sulphate (0.1 M), placed in a 20-ml glass scintillation vial, 2 ml of magnesium sulphate (0.017 M) added, and then dried at 60 °C. POC was quantified using a TC analyser (Shimadzu TOC-V Analyser & SSM-5000A Solid Sample Combustion Unit), PN by the method of Bronk and Ward (2000), and PP by the method of Solorzano and Sharp (1980).

### Inorganic carbon fixation-response curves

The dependencies of CO_2_ fixation on CO_2_ and HCO_3_^–^ were determined from experiments that involved varied TIC with fixed pH and varied pH with fixed TIC (see Supplementary Information SII, SIII) in TRIS-buffered YBCII medium using the ^14^C uptake technique (Steemann Nielsen and Jensen, 1957).

Prior to each experiment, 1 l of bicarbonate-free YBCII medium was aerated overnight with CO_2_-free air (soda-lime column). A 200-ml sample from each culture was gravity-filtered onto a 47-mm cyclopore filter (1-µm pore; Whatman 60750) and gently re-suspended into 50 ml of the CO_2_-free YBCII medium. Exactly 5 ml of concentrated culture was pipetted into each tube of the TIC or pH gradients (35 ml total volume per tube) and gently inverted to evenly distribute the trichomes. The remaining culture was used for measurement of initial activity, *T*_0_. Three replicate cultures were used per treatment. During sample preparation, test-tubes were maintained at growth temperature (26 °C) and a low light intensity (<10 µmol photons m^–2^ s^–1^).

To characterise the Ci chemistry, exactly 20 ml of culture from each treatment was filtered through a Swinnex filter (25 mm, 0.45-µm pore, glass-fibre filter): 15 ml into a plastic centrifuge tube (no headspace) for TIC analysis (Shimadzu TOC-V Analyser & ASI-V Autosampler), and 5 ml into a plastic cryogenic vial (Sigma-Aldrich V5257-250EA; no headspace) for pH analysis.

To measure chlorophyll *a* concentrations, a 1-ml sample from each treatment was pipetted into 9 ml of 100% acetone and left in a freezer (–20 °C) overnight (Welschmeyer, 1994). The sample was vortex-mixed and left in the dark (~30 min) to allow cell debris to precipitate and the solution to equilibrate to room temperature. A 2-ml aliquot was used to measure *F*_o_ using a FRRfII FastAct Fluorometer System (Chelsea Technologies Group Ltd, UK) with the same parameters as used for live cultures. Chlorophyll *a* concentrations were calculated from a calibration curve derived from a dilution series measured using a chlorophyll *a* standard (Sigma-Aldrich C5753).

To assess whether cells had been affected by concentration via filtration and re-suspension and exposure to the range of TIC and pH gradients over the course of the ^14^C incubations, 2-ml aliquots of culture from each treatment were dark-acclimated (~20 min) and the photosynthetic efficiency of PSII (*F*_v_/*F*_m_) was determined using a FRRfII FastAct Fluorometer System (Chelsea Technologies Group Ltd, UK) (see Supplementary Fig. S1).

Finally, 10 ml of culture from each treatment was pipetted into 12-ml glass (PTFE-capped) test-tubes and used for ^14^C incubations. A ^14^C spike solution was prepared by pipetting 45 µl of a ^14^C-labelled sodium bicarbonate solution (NaH^14^CO_3_) with a specific activity of 52 mCi mmol^–1^ (Perkin Elmer, USA) into 8 ml of bicarbonate-free YBCII media. Exactly 250 µl of the spike was added to each tube culture. The *T*_0_ tubes were immediately filtered through Swinnex filters containing 25-mm diameter (0.45-µm pore) glass-fibre filters, placed in scintillation vials, and acidified (500 µl of 3 M HCl). To determine the total activity (TC), 20 µl of the spike was added into three scintillation vials already containing 4.5 ml of scintillation cocktail (Gold LLT) and 200 µl of phenylethylamine. The TC vial caps were screwed tight immediately. The spiked test-tubes were placed within a custom-made water-jacketed incubator and maintained at 26 °C under saturating light intensity (400 ± 6 µmol photons m^–2^ s^–1^) (The Optoelectronic Manufacturing Corporation Ltd. 1ft T5 Daylight, UK). The incubations lasted between 60 and 90 min and took place between 4 to 6 h into the light period of the L/D cycle. The ^14^C incubations were repeated in the dark, using black-coated (Plasti-Kote paint) test-tubes. Dark ^14^C uptake rates were 8.25% (±0.46) and 7.05% (±0.25) of the maximum light-saturated ^14^C uptake rates for the TIC and pH response curves, respectively. Dark ^14^C uptake rates exhibited no response to varying TIC or pH and were used to correct the light-dependent rates of photosynthesis (Li and Dickie, 1991).

To terminate ^14^C uptake, samples were filtered through 25-mm (0.45-µm pore) glass-fibre filters (Fisherbrand FB59451, UK) using a bespoke 30-funnel filtration manifold. Test-tubes and filters were rinsed twice with 5 ml of YBCII media, before the filters were placed into scintillation vials. The vials were acidified (500 µl of 3 M HCl) overnight along with the *T*_0_ samples. Exactly 4.5 ml of scintillation cocktail (Gold LLT) was added to the acidified vials and the caps tightened. Ensuring that the scintillation cocktail and filtered samples were well mixed, the vials were placed within a scintillation counter and the disintegrations per minute (DPM) of each vial were measured (20 min per vial). The CO_2_ fixation rates were calculated using the following equation:

$$\text{C}-\text{fixation}=\left(\frac{{\text{DPM}}_{\text{S}}-{\text{DPM}}_{\text{T}0}}{{\text{DPM}}_{\text{TC}}}\right)\times \left(\frac{{\text{Vol}}_{\text{TC}}}{{\text{Vol}}_{\text{s}}}\right)\times \left(\frac{\text{TIC}}{t}\right)\times 1.05$$ (1)

where DPM_S_, DPM_T0_, and DPM_TC_ are the measurements for the sample, initial activity, and total activity vials, respectively; TIC (mmol l^–1^) is the mean concentration of total dissolved inorganic carbon within the sample over the course of the incubation (inclusive of the NaH^14^CO_3_ spike); Vol_TC_ and Vol_S_ are the volumes of the sample and TC vials, respectively; *t* is the experimental incubation time (h); and 1.05 is the radioisotope discrimination factor (^12^C:^14^C). Note that mean T_0_ and TC values were used when calculating the C-fixation rates (*n*=3).

Inorganic carbon fixation rates were normalised to a POC basis and the CO_2_ response curves were fitted to a Michaelis–Menten function:

$$V_{\text{C}}=\frac{\left(V_{\text{C},\text{max}}\;\cdot \;\left[CO_{2}\right]\right)}{\left(K_{\text{m}}\;+\;\left[CO_{2}\right]\right)}$$ (2)

where *V*_C_ is the organic C-specific rate of CO_2_ fixation, *V*_C,max_ is the maximum rate of CO_2_ fixation, and *K*_m_ is the half-saturation constant. Curve-fitting was performed on individual replicates to calculate mean (±SE) curve-fit parameters (Sigmaplot 11.0), as well on the combined data where all replicates of the varied TIC (fixed pH) and varied pH (fixed TIC) data were combined per CO_2_ treatment.

### Spectrophotometric chlorophyll *a* analysis

Samples for spectrophotometric determination of chlorophyll *a* were collected together with each CO_2_-response curve and were used to normalise productivity rates as well as to calculate the ratio of Chl *a*:C (i.e. total C). A 100-ml sample from each culture was vacuum-filtered onto a 25-mm (0.45-µm pore) glass-fibre filter (Fisherbrand FB59451, UK) and extracted in 5 ml of 100% methanol. The filters were homogenised and extracted overnight at –20 °C before being centrifuged at 12 000 *g* for 10 min and a 3-ml aliquot of the supernatant was transferred to a quartz cuvette. The absorption spectrum (400–800 nm) was measured using a spectrophotometer (Hitachi U-3000, Japan) and the Chl *a* concentration (µg l^–1^) was calculated using the following equation (Ritchie, 2008);

$$\text{Chl}\;a=\left(\frac{12.9447\times \left({\text{Abs}}_{665}-{\text{Abs}}_{750}\right)\times {\text{Vol}}_{\text{E}}}{{\text{Vol}}_{\text{F}}}\right)\times 1000$$ (3)

where Abs_665_ and Abs_750_ are the baseline-corrected optical densities of the methanol extracted sample at 665 and 750 nm, respectively; Vol_E_ is the volume of the solvent used for extraction (i.e. 5 ml); Vol_F_ is the volume of culture that was filtered (i.e. 100 ml); and 12.9447 is a cyanobacteria-specific Chl *a* coefficient for 100% methanol extraction.

### Modelling the CCM

The CO_2_ and HCO_3_^–^ fluxes and concentrations in an idealised *Trichodesmium* cell were calculated using the numerical model from Mangan *et al.* (2016) and Mangan and Brenner (2014). The aim was to provide a qualitatively informative view of the CCM system, without attempting to match carboxylation rates or fluxes to the experimental system or to rescale the results from the idealised cell to what would be expected from the experimental data. With the exception of a few key parameter values (Table 1), the model used was equivalent to that reported in Mangan *et al.* (2016). The main changes between the idealised *Trichodesmium* cell and previous models were an increase in cell and carboxysome size to be consistent with reported values for *T. erythraeum*, changes to the Rubisco kinetic constants, use of pH and external CO_2_ and HCO_3_^–^ concentrations similar to those in the ^14^C incubations, updating the pKa_eff_ for HCO_3_^–^ to CO_2_ to match that used in the CO2SYS calculation, and re-calculating the HCO_3_^–^ uptake rate to support internal inorganic carbon concentrations of ~30 mM. We scaled the Rubisco concentration by the carboxysome volume, so that the activity per volume remained the same. Similarly, we scaled the amount of carbonic anhydrase by the carboxysome surface area, so that the activity per area remained the same. The carbonic anhydrase activity was sufficient to equilibrate CO_2_ and HCO_3_^–^ to $K{´}_{\text{eq}}=[{\text{HCO}}_{{\text{3}}^{-}}/[{\text{CO}}_{\text{2}}]=\text{1}0^{–{\text{pKa}}_{e\text{ff}}}{}^{+\text{pH}}$. We set the carbonic anhydrase *K*_ca_ value to preserve the correct equilibrium value for the internal pH.

**Table 1.** Key parameter values used in the numerical simulation of the CCM in *Trichodesmium*

Variable	Units	Model value
Cell radius, *R*_b_	µm	3
Carboxysome radius, *R*_c_	µm	0.15
Rubisco reaction rate, *k*_Rub_	s^–1^ per active site	1.92
Rubisco *K*_CO2_	µM	145
Rubisco *K*_O2_	µM	600
Rubisco specificity, *S*	–	45
Number of Rubisco active sites	–	54000
Number of carbonic anhydrase active sites	–	900
Carbonic anhydrase half-maximum constant for CO_2_, *K*_ca_	µM	104.7
Internal pH	–	8.3
pKa_eff_ for HCO_3_^–^:CO_2_	–	5.84
Carboxysome permeability	cm s^–1^	3 × 10^–5^
HCO_3_^–^ uptake velocity, *j*_c_	cm s^–1^	2.4 × 10^–7^
CO_2_ to HCO_3_^–^ conversion at membrane	cm s^–1^	0.6 × 10^–7^

The cell radius was measured from a bioimage collected using fluorescence microscopy (Supplementary Fig. S12). Kinetic constants of Rubisco carboxylation (*K*_CO2_), oxygenation (*K*_O2_), and the specificity factor (*S*) for a form 1B cyanobacteria were taken from Badger *et al.* (1998).

## Results

### Inorganic carbon chemistry, growth rate, and cell composition

Overall, the CO_2_ drawdown in the cultures ranged between 57–78 µmol mol^–1^ for all CO_2_ treatments (Table 2) and exhibited a negligible CO_2_ drift over a diurnal cycle (see Supplementary Fig. S2). Dissolved inorganic NH_4_^+^ concentrations in the growth medium were ~1 µM, while NO_3_^–^ concentrations were ~0.3 µM, which was below the 1 µM detection limit.

**Table 2.** The growth conditions (±SE) achieved for *T. erythraeum* IMS101 when cultured at three target gas-phase CO_2_ concentrations (Low=180 µmol mol^–1^, Mid=380 µmol mol^–1^, and High=720 µmol mol^–1^), saturating light intensity (400 µmol photons m^–2^ s^–1^), and optimal temperature (26 °C)

Variable	Units	Low CO_2_	Mid CO_2_	High CO_2_
pH	–	8.458	8.174	7.906
H^+^	nM	3.5 (0.20)	6.7 (0.13)	12.4 (0.28)
A_T_	µM	2431 (70)	2447 (54)	2442 (56)
TIC	µM	1800 (69)	2039 (46)	2201 (50)
HCO_3_^–^	µM	1362 (67)	1743 (39)	2005 (44)
CO_3_^2–^	µM	435 (16)	289 (9)	179 (6)
CO_2_	µM	3.3 (0.3)	8.1 (0.2)	17.3 (0.5)
NH_4_^+^	µM	1.03 (0.14)	1.00 (0.08)	1.08 (0.06)
NO_3_^–^	µM	0.34 (0.05)	0.32 (0.03)	0.30 (0.02)
*n*		89	67	39

Individual pH values were converted to a H^+^ concentration, allowing a mean pH value (Total scale) to be calculated. Dissolved inorganic NH_4_^+^ was determined using the phenol-hypochlorite method as described by Solorzano (1969), while dissolved inorganic NO_3_^–^ was determined using the spectrophotometric method as described by Collos *et al.* (1999).

Balanced growth rates increased from ~0.2 d^–1^ at low CO_2_ to ~0.34 d^–1^ at mid-CO_2_ and ~0.36 d^–1^ at high CO_2_ (Table 3). The dark-adapted photochemical efficiencies of PSII (*F*_v_/*F*_m_) were proportionate to the CO_2_ treatment, increasing from 0.27 at low CO_2_ to ~0.31 at mid-CO_2_ and ~0.34 at high CO_2_ (Table 3). The particulate C:N ratio was independent of CO_2_, while the C:P and N:P ratios increased with increasing CO_2_ (Table 3). Both Chl *a*:C and Chl *a*:N ratios were about 30–40% higher at mid-CO_2_ than at low or high CO_2_.

**Table 3.** The mean (±SE) balanced growth rate, dark-adapted photochemical efficiency of PSII (*F*_v_/*F*_m_), elemental stoichiometry, and chlorophyll *a* to C and N ratios for *T. erythraeum* IMS101 when acclimated to three target CO_2_ concentrations (Low=180 µmol mol^–1^, Mid=380 µmol mol^–1^, and High=720 µmol mol^–1^), saturating light intensity (400 µmol photons m^–2^ s^–1^), and optimal temperature (26 °C)

Variable	Units	Low CO_2_	Mid CO_2_	High CO_2_
Growth rate	d^–1^	0.198 (0.027)^A^	0.336 (0.026)^B^	0.361 (0.020)^B^
*F*_v_/*F*_m_	dimensionless	0.274 (0.025)^A^	0.305 (0.020)^B^	0.342 (0.037)^C^
Elemental stoichiometry				
C:N	mol:mol	7.9 (0.8)	7.8 (0.3)	7.3 (0.8)
C:P	mol:mol	91.9 (6.3)^A^	143.6 (6.3)^B^	155.5 (13.5)^B^
N:P	mol:mol	11.9 (0.6)^A^	18.4 (0.7)^B^	21.8 (1.7)^B^
Chl *a*:C	g:mol	0.052 (0.003)^A^	0.089 (0.003)^C^	0.066 (0.003)^B^
Chl *a*:N	g:mol	0.401 (0.037)^A^	0.693 (0.035)^B^	0.474 (0.043)^A^

Replicates comprised *n*=9 at low CO_2_, *n*=6 at mid- and high CO_2_. Letters indicate significant differences between CO_2_ treatments (one-way ANOVA, Tukey *post hoc* test; *P*<0.05); where B is significantly greater than A, and C is significantly greater than B and A.

### CO_2_-response curves

**Fig. 1.**
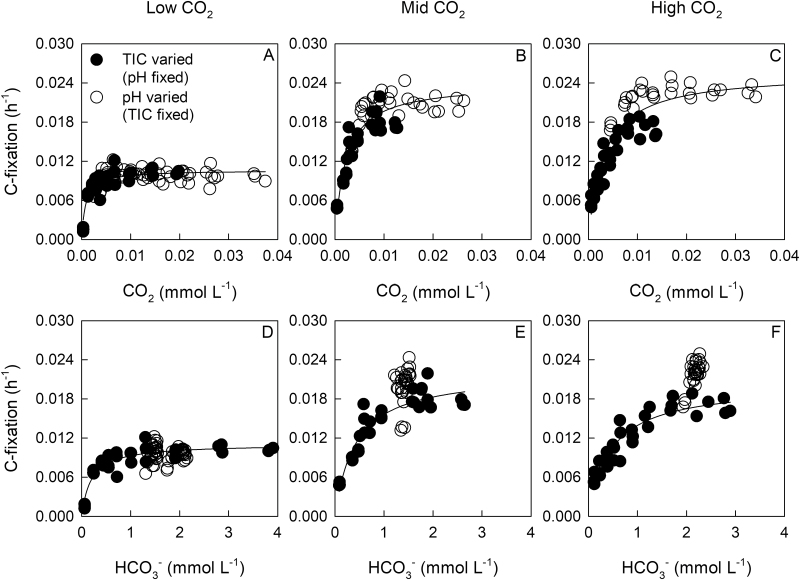
(A–C) CO_2_- and (D–F) HCO_3_^–^-response curves for inorganic C-fixation by *T. erythraeum* IMS101. C-fixation rates are normalised to a carbon h^–1^ basis. Filled circles indicate data obtained by varying TIC and HCO_3_^–^ at a fixed pH of ~8.15. Open circles indicate data obtained by varying pH (~7.52–8.54) at a fixed TIC. Differences in the range of HCO_3_^–^ and CO_2_ gradients between CO_2_ treatments were due to variability in pipetting and not from instability in the Ci chemistry. For the CO_2_ response, curve-fitting was performed using all replicates from both the TIC and pH gradients. For the HCO_3_^–^ response, curve-fitting was performed using data from the TIC gradient only. The CO_2_- and HCO_3_^–^- response curves for individual experiments are shown in Supplementary Figs S6–S11.

Based on the shape of the response curves, the inorganic carbon (^14^C) fixation rate was fitted to a saturating function of the dissolved CO_2_ concentration in both the pH gradient and TIC gradient experiments (Fig. 1). Although a saturating function of HCO_3_^–^ concentration was observed when TIC was varied at constant pH (Fig. 1A–C), Ci assimilation could not be described by the same kinetic constants when pH was varied at constant TIC (Fig. 1D–F).

The *K*_m_ for photosynthetic C-fixation increased from 0.8 µM in cultures acclimated to low CO_2_ to 2.2 µM and 3.2 µM in cultures acclimated to mid- and high CO_2_, respectively, and were approximately 4- to 5-fold lower than the ambient CO_2_ concentrations in the cultures. The maximum organic carbon-specific rate of C-fixation (*V*_C,max_) was also higher in cells grown at mid-CO_2_ than at low CO_2_, although the rates at mid- and high CO_2_ did not differ significantly (Table 4). The affinity for CO_2_ (*V*_C,max_/*K*_m_) declined by about 40% between the low- and high-CO_2_ treatments (Table 4).

**Table 4.** The physiological parameters (±SE) of the C-specific C-fixation versus CO_2_ concentration response curves for *T. erythraeum* IMS101, fitted using the Michaelis–Menten model to obtain estimates using the combined data from all replicates from both experiments employing varied TIC at fixed pH and varied pH at fixed TIC for each CO_2_ treatment

Parameter	Units	Low CO_2_	Mid CO_2_	High CO_2_
*V*_C,max_	h^–1^	0.011 (0.0002)	0.024 (0.0007)	0.026 (0.0008)
*K*_m_	µM CO_2_	0.8 (0.1)	2.2 (0.3)	3.2 (0.4)
Affinity	mM (CO_2_)^–1^ h^–1^	13.3 (1.7)	10.9 (1.5)	8.0 (1.0)

*V*_C,max_, the C-specific maximum C-fixation rate; *K*_m_, the half saturation constant; Affinity, the C-specific initial slope of the *V*_C,max_ versus CO_2_-response curve.

### Modelled response curves

**Fig. 2.**
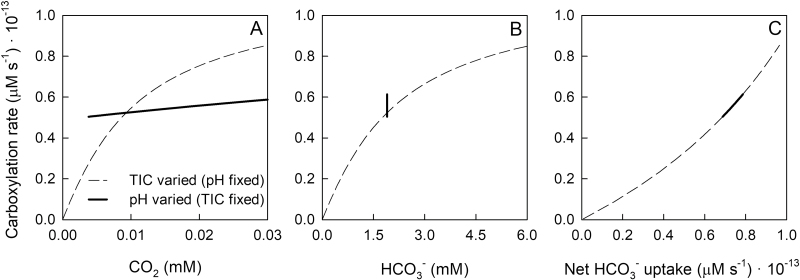
Calculated carboxylation rates obtained from model simulations for *T. erythraeum* IMS101 as a function of external CO_2_ (A) and HCO_3_^–^ (B) concentrations, with TIC (i.e. HCO_3_^–^) varied at a fixed pH=8.15 (dashed lines) and pH varied at a fixed HCO_3_^–^=1.9 mM (solid lines). Carboxylation rates are also plotted against the net HCO_3_^–^ uptake rate (C), where HCO_3_^–^ and CO_2_ leakage rates were subtracted from the rate of gross HCO_3_^–^ transport.

Without parameter-fitting, the CCM model of *Trichodesmium* produced behaviors consistent with the experimental data when either external TIC (i.e HCO_3_^–^) was varied at a fixed pH or when pH was varied at a fixed TIC (Fig. 2A, B). Assuming HCO_3_^–^ is the dominant form of inorganic carbon taken up by the cell (Kranz *et al.*, 2009; Eichner *et al.*, 2015), *Trichodesmium* exhibited a significant response to changes in external pH and CO_2_ concentrations. The decrease in carboxylation rate with decreasing external CO_2_ was due to a decrease in HCO_3_^–^ uptake (when TIC was varied) or an increase in HCO_3_^–^ and CO_2_ leakage out of the cell (when pH was varied) (Supplementary Fig. S3). Modelled carboxylation rates from both numerical simulations exhibited a smooth function of HCO_3_^–^ uptake, HCO_3_^–^ leakage, and CO_2_ leakage (Fig. 2C).

The *V*_C,max_ of the pH gradient and TIC gradient experiments were not significantly different (Supplementary Table S3). However, the maximum carboxylation rates from the simulations were significantly different (Fig. 2); principally because the external HCO_3_^–^ concentration used in the pH-dependent simulation (chosen to be the same as the experiment) was not sufficient to saturate Rubisco. It is possible that the *K*_m_ value assumed for Rubisco was set too high, or the internal pH, geometry, or HCO_3_^–^ uptake values were substantially different. Note that we were simulating values beyond the range of those in the experiments, so such a discrepancy is magnified.

## Discussion

The key findings of our study were as follows. The acclimated growth rate increased from low- to mid-CO_2_ treatments but did not increase significantly between mid- and high-CO_2_, suggesting that the positive effect of elevated CO_2_ on *Trichodesmium* carbon assimilation over the coming decades may only be slight. The maximum rate (*V*_C,max_) and the half-saturation constant (*K*_m_) for C-fixation increased with increasing CO_2_ treatment, but the affinity for CO_2_ (*V*_C,max_/*K*_m_) declined, which is probably attributable to the activity of the CCM in *Trichodesmium*. The measured inorganic C-fixation rate in *Trichodesmium* could be described as a saturating function of CO_2_, both when CO_2_ was manipulated by varying pH at constant TIC and when CO_2_ was manipulated by varying TIC at constant pH. A mechanistic model of the CCM in *Trichodesmium* indicated that the former was due to HCO_3_^–^ uptake limitation of carboxylation rate, whereas the latter was due to a high-pH-mediated increases in HCO_3_^–^ and CO_2_ leakage, potentially coupled to other unknown processes operating outside of the paramaterised model that were restricting Ci assimilation rates at high pH. Such processes may involve the direct effect of pH on membrane conformation, membrane transport processes, or metabolic functions.

### Effect of acclimation to variations in inorganic chemistry on growth rates and elemental stoichiometry

The increased growth rates that we observed from low- (180 µmol mol^–1^) to mid- (380 µmol mol^–1^) and high-CO_2_ treatments (720 µmol mol^–1^) were similar to previous findings (Barcelos e Ramos *et al.*, 2007; Boatman *et al.*, 2017, 2018*b*). The growth rate at high CO_2_ was 8% greater than at mid-CO_2_, but this difference was not statistically significant. The magnitude of this increase at high CO_2_ was comparable to several recent studies, which report growth rate increases of 7–26% with increases of CO_2_ beyond 400 µmol mol^–1^ (Barcelos e Ramos *et al.*, 2007; Hutchins *et al.*, 2007; Levitan *et al.*, 2007; Kranz *et al.*, 2010; Garcia *et al.*, 2011; Boatman *et al.*, 2017).

The observed increases in C:P and N:P were consistent with previous findings (Barcelos e Ramos *et al.*, 2007; Kranz *et al.*, 2010; Levitan *et al.*, 2010), with changes that can be ascribed to increases in cellular N and C incorporation, with P content relatively unaffected by CO_2_ (Hutchins *et al.*, 2007; Kranz *et al.*, 2010). In contrast, the C:N ratio and thus the balance between CO_2_ fixation and N_2_ fixation was not significantly affected by the CO_2_ treatment. Similarly, Levitan *et al.* (2007) found that C:N varied only slightly (from 6.5 to 7.0) across growth CO_2_ concentrations ranging from 250 to 900 µmol mol^–1^.

We report C-specific rates here as these are most directly related to changes in specific growth rate because both rates can be expressed in equivalent units of inverse time (e.g. h^–1^ or d^–1^). However, we note that due to differences in the Chl *a*:C ratio, chlorophyll *a*-specific rates showed a different pattern, increasing progressively from low through mid- to high CO_2_ (see Supplementary Table S2, Supplementary Fig. S4). A reduction in Chl *a*:C decreases the energy demands associated with synthesis of the photosynthetic apparatus and is dictated by the total demands for reductant (NADPH) and high-energy phosphate bonds (ATP) (Geider *et al.*, 2009), the minimum turnover times for PSII (τ_PII_ʹ) and PSI (τ_PI_ʹ), and the minimum pigment content required for effective light absorption and energy transfer (*a*_min_ʹ) (Behrenfeld *et al.*, 2008). We suggest that the reduced Chl *a*:C at low CO_2_ relative to mid-CO_2_ was probably due to the cost of up-regulating the CCM, whereas the reduced Chl *a*:C at high CO_2_ may have been due to an increase in carbohydrate storage granules relative to the mid-CO_2_ treatment (Table 3).

### CO_2_-response curves

The growth rates reported here were comparable to the 2-µM EDTA, iron-replete (unchelated) treatments in Boatman *et al.* (2017), as well as 20-µM EDTA, iron-replete (chelated) cultures (Boatman *et al.*, 2018*b*), which suggests that our cultures were not exposed to toxic concentrations of certain trace metals (e.g. copper) caused from low trace metal buffering capacity, as reported by Hong *et al.* (2017). Furthermore, dissolved inorganic NH_4_^+^ concentrations were consistently around 1.0 µM (Table 2). We are therefore confident that the observed positive effect of ocean acidification on growth and primary productivity is driven by the increased CO_2_ concentration, rather than being a consequence of a pH-induced shift of the NH_3_/NH_4_^+^ equilibrium. We determined CO_2_-response curves at one time of day (4–6 h into the photoperiod of a 12/12 h L/D cycle) and as such cannot extrapolate to a diel response given the reports of temporal separation of photosynthesis and N_2_ fixation in *Trichodesmium* (Berman-Frank *et al.*, 2001).

The mechanistic model of Mangan *et al.* (2016) indicates that the CO_2_ response we observed when the TIC was varied (pH fixed) was caused by HCO_3_^–^ limitation, where HCO_3_^–^ uptake limits the rate of carboxylation. Conversely, the CO_2_ response we observed when pH was varied (TIC fixed) was a function of the pH dependency of HCO_3_^–^ and CO_2_ leakage, which in turn could lead to CO_2_ limitation of C-fixation and/or diversion of reducing equivalents from powering CO_2_ fixation via the Calvin cycle to powering the conversion of CO_2_ to HCO_3_^–^ by the NDH-I_4_ complex. The model of the CCM in *Trichodesmium* showed the relative importance of leakage, which is notably sensitive to certain parameters in the system such as internal pH, Rubisco activity, cell size, and carboxysome size.

Previous studies have shown a notable response in CCM activity to changes in CO_2_; for example, a two-fold lower dissolved inorganic carbon half-saturation concentration in cells acclimated to 150 µmol mol^–1^ (pH 8.56) compared with 370 µmol mol^–1^ (pH 8.26) (Kranz *et al.*, 2009). Our experimental observations indicated that Ci assimilation (*V*_C_) was well described by a CO_2_-response curve, but not by a single HCO_3_^–^-response curve (Fig. 1). We now offer an explanation as to the response of *V*_C_ to HCO_3_^–^ concentration in the experiments where we varied pH from 7.65 to 8.5 at constant TIC.

Based on the numerical simulations, carboxylation rates across an external pH gradient ranging from 7.5 to 8.5 exhibited a clear linear response, which could not be ascribed to a Michaelis–Menten function (see Supplementary Fig. S3). Conversely, our experimental data showed a clear and significant decrease in Ci assimilation rates at low external CO_2_/high pH (Fig. 1). In addition, the Ci assimilation rates for the pH-gradient and TIC-gradient experiments, for all replicates of all three CO_2_ treatments, exhibited similar inflection points to external CO_2_ (Supplementary Fig. S5). In order for the simulated system to exhibit a rate-saturating response to external CO_2_, CO_2_ would have to be the dominant source of inorganic carbon. This would contradict all previous research showing that HCO_3_^–^ accounts for >90% of inorganic carbon uptake (Kranz *et al.*, 2009, 2010) and the currently accepted mechanism of Ci assimilation in *T. erythraeum* IMS101 (Badger and Price, 2003).

Given how well the numerical simulations modelled carboxylation rates as a smooth function of HCO_3_^–^ uptake, HCO_3_^–^ leakage, and CO_2_ leakage (Fig. 2C), we propose that the linear pH-dependency of carboxylation rate predicted by the model is mechanistically correct, but that processes not captured by the model are contributing to the decrease in Ci assimilation rate at high pH. Such factors could include a direct effect of high pH on cell membrane properties and alteration in membrane conformation (Myklestad and Swift, 1998), or the influence of pH on membrane transport processes and metabolic functions involved in cellular pH regulation (Raven, 1981).

Interestingly, for the mid- and high-CO_2_ treatments, a Michaelis–Menten function provided a better fit for the pH-varied (TIC fixed) data than a linear regression. However, there was no significant difference between a linear or Michaelis–Menten function for the low-CO_2_ data, which suggests that full acclimation to a high-pH environment prior to the ^14^C incubations lessened the negative effect that high pH had on Ci assimilation.

Based on our simulation, the actual carboxylation rate of *Trichodesmium* should be modelled as a function of HCO_3_^–^ and pH. This is because the CO_2_ concentration in a saturated HCO_3_^–^/high-pH environment (i.e. 3.8 mM HCO_3_^–^, pH=8.4) could be equivalent to a limited HCO_3_^–^/present-day pH environment (i.e. 1.9 mM HCO_3_^–^, pH=8.1); which for the aforementioned reasons will impose different constraints on leakage/uptake rates. That said, our experimental data clearly suggested that high-pH-induced processes operating outside of the CCM were contributing to decrease Ci assimilation. Overall, this may allow the Ci assimilation rates of *Trichodesmium* to be ascribed as a function of CO_2_ (Fig. 1, see Supplementary Fig. S4), which would be considerably simpler to implement in biogeochemical models of *Trichodesmium* growth and photosynthesis (Hutchins *et al.*, 2013) than a HCO_3_^–^-response curve in which the kinetic constants (*K*_m_ and *V*_m_) are pH-dependent. Further experimental work is needed to assess whether a CO_2_ parameterisation is consistent across a more extended range of pH and HCO_3_^–^ conditions than those used in our experiments.

## Conclusions

Climate change is driving ocean acidification, which results in higher CO_2_ and HCO_3_^–^ concentrations and a decrease in pH. We observed systematic changes in the kinetics of inorganic carbon assimilation of *T. erythraeum* IMS101 in response to acclimation to increasing CO_2_ concentrations ranging from low CO_2_ (levels at the last glacial maximum) through mid-CO_2_ (levels at the end of the 20th century), to high CO_2_ (levels predicted for 2050–2100). Extrapolating these responses to future scenarios of the natural environment should take into account the fact that our findings were obtained using acclimation experiments whereas *Trichodesmium* may adapt to future conditions (Hutchins *et al.*, 2015), that variability may exist between strains and clades (Hutchins *et al.*, 2013), and that there will be additional effects of integrated abiotic variables (e.g. light and temperature) and nutrients (e.g. P and Fe) on *Trichodesmium* productivity (Walworth *et al.*, 2016; Boatman *et al.*, 2018*a*, 2018*b*).

In the context of the open oceans, our results indicate that nutrient-replete net photosynthesis and growth rates of *T. erythraeum* IMS101 would have been severely CO_2_-limited at the last glacial maximum relative to current conditions. However, future increases in CO_2_ (i.e. 720 µmol mol^–1^) may not significantly increase its growth and productivity, although we note that other studies have reported a stimulation of growth and photosynthesis by increasing CO_2_ beyond current ambient concentrations (Hutchins *et al.*, 2007; Levitan *et al.*, 2007, 2010). On the other hand, we did observe that growth under high CO_2_ will increase key stoichiometric ratios (N:P and C:P). Increases of N:P and C:P in *Trichodesmium*-dominated oceanic regimes may affect bacterial and zooplankton metabolism, the pool of bioavailable nitrogen, the depth at which sinking organic matter is remineralised, and consequently carbon sequestration via the biological carbon pump (Mulholland *et al.*, 2004; McGillicuddy, 2014). These responses could serve as a negative feedback to climate change by increasing new N and C production and thereby increasing the organic carbon sinking to the deep ocean.

## Supplementary data

Supplementary data are available at *JXB* online.

Information SI. Calculation of inorganic carbon speciation.

Information SII. Preparation of medium for CO_2_-response curves where TIC was varied at fixed pH.

Information SIII. Preparation of medium for CO_2_-response curves where pH was varied at fixed TIC.

Table S1. Recent literature on the C- and N_2_-fixation rates and elemental stoichiometry of *T. erythraeum* IMS101 in response to CO_2_, temperature, and light.

Table S2. The Chl *a*-specific curve-fitting parameter values of the carbon assimilation–CO_2_-response curves when the ‘TIC varied/pH fixed’ and ‘pH varied/TIC fixed’ data were modelled separately.

Table S3. The carbon-specific curve-fitting parameter values of the carbon assimilation–CO_2_-response curves when the ‘TIC varied/pH fixed’ and ‘pH varied/TIC fixed’ data were modelled separately.

Fig. S1. The effect of filtration/re-suspension and incubation on photosynthetic efficiency.

Fig. S2. The inorganic carbon chemistry of the culture vessels over a diurnal period.

Fig. S3. The modelled rates of carboxylation, CO_2_ leakage, HCO_3_^–^, uptake and HCO_3_^–^ leakage for a *Trichodesmium* cell.

Fig. S4. The Chl *a*-specific curve fits of the carbon assimilation–CO_2_-response curves when the ‘TIC varied/pH fixed’ and ‘pH varied/TIC fixed’ data were modelled together.

Fig. S5. The Chl *a*- and carbon-specific curve fits of the carbon assimilation–CO_2_-response curves when the ‘TIC varied/pH fixed’ and ‘pH varied/TIC fixed’ data were modelled seperately.

Fig. S6. The Chl *a*- and carbon-specific curve fits of the carbon assimilation–CO_2_-response curves of the low-CO_2_ treatment for the ‘TIC varied/pH fixed’ data.

Fig. S7. The Chl *a*- and carbon-specific curve fits of the carbon assimilation–CO_2_-response curves of the low-CO_2_ treatment for the ‘pH varied/TIC fixed’ data.

Fig. S8. The Chl *a*- and carbon-specific curve fits of the carbon assimilation–CO_2_-response curves of the mid-CO_2_ treatment for the ‘TIC varied/pH fixed’ data.

Fig. S9. The Chl *a*- and carbon-specific curve fits of the carbon assimilation–CO_2_-response curves of the mid-CO_2_ treatment for the ‘pH varied/TIC fixed’ data.

Fig. S10. The Chl *a*- and carbon-specific curve fits of the carbon assimilation–CO_2_-response curves of the high-CO_2_ treatment for the ‘TIC varied/pH fixed’ data.

Fig. S11. The Chl *a*- and carbon-specific curve fits of the carbon assimilation–CO_2_-response curves of the high-CO_2_ treatment for the ‘pH varied/TIC fixed’ data.

Fig. S12. A bioimage of *T. erythraeum* IMS101 filaments cultured at mid-CO_2_, saturating light, and optimal temperature.

Supplementary Tables and Figures
