# Supplementary material for: Inorganic carbon and pH dependency of photosynthetic rates in Trichodesmium
Source: J Exp Bot. 2018 Apr 12;69(15):3651–60. doi: 10.1093/jxb/ery141 (PMC6022602; doi:10.1093/jxb/ery141)
Supplement: Supplementary Tables and Figures [file ery141_suppl_supplementary_tables_figures.pdf]

Supplementary Information for

Inorganic carbon and pH dependency of *Trichodesmium's* photosynthetic rates.

TOBIAS G. BOATMAN*, NIALL M. MANGAN, TRACY LAWSON, RICHARD J. GEIDER

*Address correspondence to tboatman@chelsea.co.uk

Contents

I. Calculation of inorganic carbon speciation.

II. Medium preparation for CO_2 response curves where TIC is varied at a fixed pH.

III. Medium preparation for CO_2 response curves where pH is varied at a fixed TIC.

IV. References.

I. Calculation of inorganic carbon speciation.

Prior to sampling, the carbonate chemistry of all cultures were measured diurnally and the inorganic carbon chemistry defined via *CO2SYS* (Lewis and Wallace, 1998) using: 1st and 2nd equilibrium constants (K1 and K2) for carbonic acid (Millero, 2010), the dissociation constant for KSO_4 (Dickson, 1990), the boric acid constant (KB) (Lee *et al.*, 2010) and the total pH. Calculated CO_2 drifts were negligible to the targeted CO_2 concentrations (e.g. 180, 380 and 720 µmol mol^{-1}), verifying that the rate of aeration was sufficient to maintain a CO_2 concentration at high cell densities (Fig. S2).

II. Medium preparation for CO_2 response curves where TIC is varied at a fixed pH.

TRIS buffered YBCII medium at pH 8.15 was prepared as follows. Exactly 3.38 mL of HCl (32%) and 4.034 g of 2-amino-2 hydroxymethyl-1,3 propanediol (TRIS, 20 mM) were added to 1 L of bicarbonate-free YBCII media and the pH adjusted to 8.15 by adding NaOH (1 M) or HCl (2 M). The bicarbonate-free media was split into two 1 L laboratory glass bottles to produce a low-TIC (600 mL) and a high-TIC (400 mL) media. To produce the high-TIC medium, exactly 0.0281 g of sodium carbonate (added first) and 0.1142 g of sodium bicarbonate (added second) were added to obtain a 5 mM TIC concentration. Different volumes of the low and high-TIC media were mixed to produce ten TIC treatments (~ 0 – 5 mmol L^{-1}), each at 90 mL total volume. Each TIC treatment was further separated into three 50 mL plastic tubes (30 mL per tube); one tube for each of the three independent cultures. The dilution series for the TIC gradient resulted in a constant pH (~ 8.15), with a range of CO_2 (~ 0 – 800 µmol mol^{-1}), CO_2 (~ 0 – 0.02 mmol L^{-1}) and HCO_3^- (~ 0 – 3.5 mmol L^{-1}) concentrations.

III. Medium preparation for CO_2 response curves where pH is varied at a fixed TIC.

TRIS buffered YBCII medium at 2.2 mM TIC was prepared as follows. Exactly 3.38 mL of HCl (32%) and 4.034 g of 2-amino-2 hydroxymethyl-1,3 propanediol (TRIS, 20 mM) were added to 1 L of bicarbonate-free YBCII media. Exactly 0.0387 g of sodium bicarbonate (added first) and 0.1571 g of sodium carbonate (added second) were added, which produced a 2.2 mM TIC concentration. The media was split into two 500 mL volumes, where the pH was adjusted by adding NaOH (1 M) or HCl (2 M) to obtain a high-pH (~ 8.5) and low-pH (~ 7.6) medium,

respectively. Different volumes of the low and high-pH media were mixed to produce ten pH treatments of varying H^+ concentrations, each at 90 mL total volume. Each pH treatment was separated into three 50 mL plastic tubes (30 mL per tube); one tube for each of the three independent cultures. The dilution series for the pH gradient resulted in a constant TIC (~ 2.2 mmol L^{-1}) and HCO_3^- concentration (~ 1.9 mmol L^{-1}) with a range of pH values (~ 7.52 – 8.54) and CO_2 (~ 0 – 800 µmol mol^{-1}) and CO_2 (~ 0 – 0.04 mmol L^{-1}) concentrations.

IV. References.

Barcelos e Ramos J, Biswas H, Schulz KG, LaRoche J, Riebesell U. 2007. Effect of rising atmospheric carbon dioxide on the marine nitrogen fixer *Trichodesmium*. *Global biogeochemical cycles* **21**, GB2028.

Breitbarth E, Oschlies A, Laroche J. 2007. Physiological constraints on the global distribution of *Trichodesmium* - effect of temperature on diazotrophy. *Biogeosciences* **4**, 53-61.

Chen Y-B, Dominic B, Mellon MT, Zehr JP. 1998. Circadian rhythm of nitrogenase gene expression in the diazotrophic filamentous nonheterocystous cyanobacterium *Trichodesmium* sp. strain IMS 101. *Journal of bacteriology* **180**, 3598-3605.

Chen YB, Zehr JP, Mellon M. 1996. Growth and nitrogen fixation of the diazotrophic filamentous nonheterocystous cyanobacterium Trichodesmium Sp. IMS 101 in defined media: evidence for a circadian rhythm. *Journal of Phycology* **32**, 916-923.

Dickson AG. 1990. Thermodynamics of the dissociation of boric acid in synthetic seawater from 273.15 to 318.15 K. *Deep Sea Research Part A. Oceanographic Research Papers* **37**, 755-766.

Eichner M, Kranz SA, Rost B. 2014. Combined effects of different CO_2 levels and N sources on the diazotrophic cyanobacterium *Trichodesmium*. *Physiologia Plantarum* **152**, 316-330.

Garcia NS, Fu F-X, Breene CL, Bernhardt PW, Mulholland MR, Sohm JA, Hutchins DA. 2011. Interactive effects of Irradiance and CO_2 on CO_2 fixation and N_2 fixation in the Diazotroph *Trichodesmium erythraeum* (Cyanobacteria). *Journal of Phycology* **47**, 1292-1303.

Hutchins D, Fu FX, Zhang Y, Warner M, Feng Y, Portune K, Bernhardt P, Mulholland M. 2007. CO_2 control of *Trichodesmium* N_2 fixation, photosynthesis, growth rates, and elemental ratios: implications for past, present, and future ocean biogeochemistry. *Limnology and Oceanography* **52**, 1293-1304.

Kranz S, Sültemeyer D, Richter KU, Rost B. 2009. Carbon acquisition in *Trichodesmium*: The effect of pCO2 and diurnal changes. *Limnology and Oceanography* **54**, 548-559.

Kranz SA, Levitan O, Richter KU, Prášil O, Berman-Frank I, Rost B. 2010. Combined effects of CO_2 and light on the N_2-fixing cyanobacterium *Trichodesmium* IMS101: physiological responses. *Plant physiology* **154**, 334-345.

Lee K, Kim T-W, Byrne RH, Millero FJ, Feely RA, Liu Y-M. 2010. The universal ratio of boron to chlorinity for the North Pacific and North Atlantic oceans. *Geochimica et Cosmochimica Acta* **74**, 1801-1811.

Levitan O, Brown CM, Sudhaus S, Campbell D, LaRoche J, Berman-Frank I. 2010. Regulation of nitrogen metabolism in the marine diazotroph *Trichodesmium* IMS101 under varying temperatures and atmospheric CO_2 concentrations. *Environmental Microbiology* **12**, 1899-1912.

Levitan O, Rosenberg G, Setlik I, Setlikova E, Grigel J, Klepetar J, Prasil O, Berman-Frank I. 2007. Elevated CO_2 enhances nitrogen fixation and growth in the marine cyanobacterium *Trichodesmium*. *Global Change Biology* **13**, 531-538.

Lewis E, Wallace D. 1998. CO2SYS Program. *Carbon Dioxide Information Analysis Center, Oak Ridge National Laboratory Environmental Sciences Division, Oak Ridge, Tennessee*.

Millero FJ. 2010. Carbonate constants for estuarine waters. *Marine and Freshwater Research* **61**, 139-142.

Mulholland MR, Capone DG. 2001. Stoichiometry of nitrogen and carbon utilization in cultured populations of *Trichodesmium* IMS101: implications for growth. *Limnology and Oceanography* **46**, 436-443.

Paerl HW, Prufert-Bebout LE, Guo C. 1994. Iron-stimulated N_2 fixation and growth in natural and cultured populations of the planktonic marine cyanobacteria *Trichodesmium* spp. *Applied and environmental microbiology* **60**, 1044-1047.

Shi D, Kranz SA, Kim JM, Morel FMM. 2012. Ocean acidification slows nitrogen fixation and growth in the dominant diazotroph *Trichodesmium* under low-iron conditions. *Proceedings of the National Academy of Sciences* **109**, 3094-3100.

Spungin D, Berman-Frank I, Levitan O. 2014. *Trichodesmium's* strategies to alleviate phosphorus limitation in the future acidified oceans. *Environmental Microbiology* **16**, 1935-1947.

Tuit C, Waterbury J, Ravizza G. 2004. Diel variation of molybdenum and iron in marine diazotrophic cyanobacteria. *Limnology and Oceanography* **49**, 978-990.

Table S1. *The current literature for* T. erythraeum IMS101, *regarding the effects of temperature (°C),* CO_2 *(µmol mol*$^{-1}$*), light intensity (µmol photons m*$^{-2}$ *s*$^{-1}$*) and light dark cycle (hr:hr) on primary productivity (*CO_2 *and* N_2 *fixation rates), elemental composition (C, N, P, Chl a) and stoichiometric ratios (C:N, C:P, N:P, Chl a:C).*

Growth Conditions				Acclimation (Generation)	μ (d^{-1})	Carbon Fixation		N_2 Fixation		Stoichiometric Ratios				Cellular Elemental Contents				Ref
**Temperature**	**Light**	**CO_2**	**L:D**							**C:N**	**C:P**	**N:P**	**Chl *a*:C**	**C**	**N**	**P**	**Chl *a***	
26	90	–	12:12	–	0.25-0.33			0.03-0.3	µmol N (µg Chl *a*)$^{-1}$ h^{-1}									Chen *et al.* (1996)
24	100	–	12:12	–	–			Up to 0.012	µmol N (µg Chol *a*)$^{-1}$ h^{-1}									Chen *et al.* (1998)
25	100	Ambient	14:10	–	–	0.22	µmol C (µg Chl *a*)$^{-1}$ h^{-1}	0.013	µmol N (µg Chl *a*)$^{-1}$ h^{-1}									Paerl *et al.* (1994)
20	100	–	12:12	15	0.04			0.060	mmol N (mol C)$^{-1}$ h^{-1} (3:1)	5.4								Breitbarth *et al.* (2007)
22					0.10			0.085										
25					0.18			0.100		6.8								
27					0.25			0.120		6.2								
30					0.21			0.095		6.0								
34					0.07			0.040		4.1								
25	150	180	12:12	7	0.36	1.75	µmol C (µg Chl *a*)$^{-1}$ d^{-1}	0.36	µmol N (µg Chl *a*)$^{-1}$ d^{-1}	4.83								Eichner *et al.* (2014)
		380			0.34	1.60		0.33		4.81								
		980			0.32	1.26		0.26		4.89								
		1400			0.27	1.62		0.33		4.93								
		180[a]		11	0.34	1.89		0.39		4.82								
		380[a]			0.37	1.82		0.39		4.68								
		980[a]			0.35	2.15		0.45		4.82								
		1400[a]			0.29	2.02		0.44		4.56								
25	80-120	250	12:12	5	0.13			1.0	nmol N (mg Chl *a*)$^{-1}$ h^{-1}	6.53								Levitan *et al.* (2007)
		400			0.16			1.3		6.52								
		900			0.26			3.1		7.04								
28	55-65	–	12:12	–	0.12	0.25	µmol C (µg Chl *a*)$^{-1}$ h^{-1}	0.015	µmol N (µg Chl *a*)$^{-1}$ h^{-1}	6.20								Mulholland and Capone (2001)
28	30	–	14:10	–	0.54			0.24-0.27	µmol N	6.20								Tuit *et al.* (2004)

									(µg Chl *a*)$^{-1}$ h^{-1} (4:1)									
25	100	380	12:12	7 - 10	0.35	1.25	mg C (mg Chl *a*)$^{-1}$ h^{-1}	14.8	µmol N (mg Chl *a*)$^{-1}$ h^{-1} (3:1)	5.10		17.7						Hutchins *et al.* (2007)
25		750			0.39	1.75		20.0		5.10		20.5						
29		380			0.36	1.24		13.5		5.20		19.0						
29		750			0.41	1.87		18.2		4.70		22.1						
25	80	400	12:12	10	0.18					7.31	138.6	19.4						Levitan *et al.* (2010)
25		900			0.32					6.43	117.1	18.4						
31		250			0.26					6.15	130.0	20.4						
31		400			0.27					6.59	161.4	25.0						
31		900			0.38					5.99	195.7	32.4						
27	90	380[b]	14:10	–	0.26	9.91	mmol C (mol C)$^{-1}$ h^{-1}	2.45	mmol N (mol C)$^{-1}$ h^{-1}	8.32			0.095					Shi *et al.* (2012)
		750[b]			0.19	8.24		1.61		7.37			0.101					
		380[c]			0.46	10.64		3.39		8.25			0.119					
		750[c]			0.37	9.58		1.70		6.85			0.116					
25	150	150-180	14:10	35	0.26			0.6-3.3	fmol N (Cell)$^{-1}$ h^{-1}	5.6	78	14		5.0	0.95	0.07	0.63	Barcelos e Ramos *et al.* (2007)
		250			0.41			2.5-3.5		5.3	85	16		4.2	0.80	0.05	0.72	
		380			0.44			2.5		5.2	95	18		4.2	0.80	0.05	0.75	
		600			0.45			1.3		5.1	105	20						
		830			0.46			1.6		5.0	120	24		1.5	0.30	0.02	0.30	
24	38	Ambient	12:12	7 - 10	0.12	0	fmol C (Cell)$^{-1}$ h^{-1}	1	fmol N (Cell)$^{-1}$ h^{-1} (3:1)					8	1.2	1.1		Garcia *et al.* (2011)
		750			0.12	0		2						9	1.2	1.5		
	100	Ambient			0.25	160		12						15	2.0	1.1		
		750			0.32	300		22						14	2.2	1.6		
	220	Ambient			0.30	200		15						15	2.0	1.2		
		750			0.38	350		20						17	2.4	1.7		
25	150	150	12:12	5	0.35		µmol C (mg Chl *a*)$^{-1}$ h^{-1}		µmol N (mg Chl *a*)$^{-1}$ h^{-1}					4.5	1.0			Kranz *et al.* (2009)
		370			0.29	51.7		11.4						4.0	0.8			
		1000			0.32	67.6		14.9						5.0	1.2			
25	50	150	–	–	0.15					6.41				3.79	0.59	0.07		Kranz *et al.* (2010)
		900			0.24					5.04				4.51	0.88	0.08		
	200	150			0.38					5.25				4.60	0.86	0.07		
		900			0.42					4.85				5.02	1.04	0.07		
25	80 - 100	400	12:12	47	0.37					8.1	90.2	11.3		18.3	2.2	0.2	1.4	Spungin *et al.* (2014)
		900			0.58					7.4	89.8	12.5		14.2	1.9	0.2	1.5	
		400[d]			0.22					10.2	184.3	14.2		18.7	1.7	0.1	0.7	
		900[d]			0.31					9.1	195.2	27.1		21.6	2.1	0.1	0.4	

Abbreviations; stoichiometric ratios are in units of mol:mol for C:N, C:P, N:P and g:mol for Chl *a*:C. Cellular elemental contents are in units of pmol $cell^{-1}$ for carbon, nitrogen and phosphorus and pg $cell^{-1}$ for Chl *a*. A dash (−) represents undefined growth conditions or acclimation time. ImageJ software was used to calculate values of productivity, stoichiometry and elemental composition from figures reported in literature. Studies which report a molar conversion ratio for acetylene to N_2 fixed (i.e. 3:1 or 4:1) are exhibited in parentheses next to the N_2 fixation rates. Superscripts in the CO_2 column indicate the following differences from the standard YBCII culture medium; ^{a} = 100 µM NO_3^-, ^{b} = 40 pM Fe´, ^{c} = 1250 pM Fe´ and ^{d} = 0.5 µM P.

Table S2. *The physiological parameters of the Chl a-specific P-CO_2 response curves for* T. erythraeum IMS101, *using two methods for manipulating the inorganic carbon chemistry (Ci). Cultures were acclimated to three CO_2 concentrations (Low = 180 µmol mol^{-1}, Mid = 380 µmol mol^{-1} and High = 720 µmol mol^{-1}), saturating light intensity (400 µmol photons m^{-2} s^{-1}) and optimal temperature (26 °C).*

Parameters	Units	Low CO_2		Mid CO_2		High CO_2	
TIC + HCO_3^- varied (pH fixed)							
V_m^{Chl}	mol C (g Chl *a*)$^{-1}$ h^{-1}	0.178 (0.007)	[A]*	0.266 (0.007)	[B]	0.344 (0.021)	[C]
K_m	µM CO_2	1.07 (0.03)	[A]*	2.03 (0.15)	[B]*	2.30 (0.35)	[B]
Affinity^{Chl}	mol C (g Chl *a*)$^{-1}$ h^{-1} (mM CO_2)$^{-1}$	167 (10)	[B]*	131 (6)	[A]*	156 (19)	*
pH varied (TIC + HCO_3^- fixed)							
V_m^{Chl}	mol C (g Chl *a*)$^{-1}$ h^{-1}	0.228 (0.006)	[A]*	0.249 (0.004)		0.339 (0.019)	[B]
K_m	µM CO_2	0.67 (0.06)	[A]*	1.47 (0.03)	[B]*	1.37 (0.12)	[B]
Affinity^{Chl}	mol C (g Chl *a*)$^{-1}$ h^{-1} (mM CO_2)$^{-1}$	358 (36)	[B]*	170 (7)	[A]*	253 (21)	*

Productivity curves were fitted using a Michaelis–Menten function to yield the greatest r^2 value (> 0.917). Letters in parenthesis indicate significant differences between CO_2 treatments (One Way ANOVA, Tukey post hoc test; $P < .05$); where [B] is significantly greater than [A] and [C] is significantly greater than [B] and [A]; an asterisk indicates a significant difference (t-test; $P < .05$) between the two Ci manipulation methods. Abbreviations; V_m^{Chl}, the Chl *a* -specific maximum C-fixation rates; K_m, the half saturation concentration; Affinity^{Chl}, the Chl *a*-specific initial slope of the V_m^{Chl} vs CO_2 curve.

Table S3. *The physiological parameters of the C-specific P-CO_2 response curves for* T. erythraeum IMS101, *using two methods for manipulating the inorganic carbon chemistry (Ci). Cultures were acclimated to three CO_2 concentrations (Low = 180 µmol mol^{-1}, Mid = 380 µmol mol^{-1} and High = 720 µmol mol^{-1}), saturating light intensity (400 µmol photons m^{-2} s^{-1}) and optimal temperature (26 °C).*

Parameters	Units	Low CO_2		Mid CO_2		High CO_2	
TIC + HCO_3^- varied (pH fixed)							
V_{max}^C	h^{-1}	0.011 (0.0003)	[A]	0.022 (0.0004)	[B]	0.020 (0.0006)	[B]*
K_m^C	µM CO_2	1.07 (0.03)	[A]*	2.03 (0.15)	[B]*	2.30 (0.35)	[B]
Affinity^C	mM $(CO_2)^{-1}$ h^{-1}	10.5 (0.5)		11.0 (0.9)	*	9.4 (1.7)	*
pH varied (TIC + HCO_3^- fixed)							
V_{max}^C	h^{-1}	0.010 (0.0003)	[A]	0.023 (0.0003)	[B]	0.024 (0.0003)	[B]*
K_m^C	µM CO_2	0.67 (0.06)	[A]*	1.47 (0.03)	[B]*	1.37 (0.17)	[B]
Affinity^C	mM $(CO_2)^{-1}$ h^{-1}	16.5 (2.0)		15.9 (0.4)	*	18.2 (1.9)	*

The r^2 values of all curve fits were > 0.917. Letters in parenthesis indicate significant differences between CO_2 treatments (One Way ANOVA, Tukey post hoc test; $P < .05$); where [B] is significantly greater than [A] and [C] is significantly greater than [B] and [A]. Abbreviations; V_m^C, the C-specific maximum C-fixation rates; K_m, the half saturation concentration; Affinity^C, the C-specific initial slope of the V_m^C vs CO_2 curve.

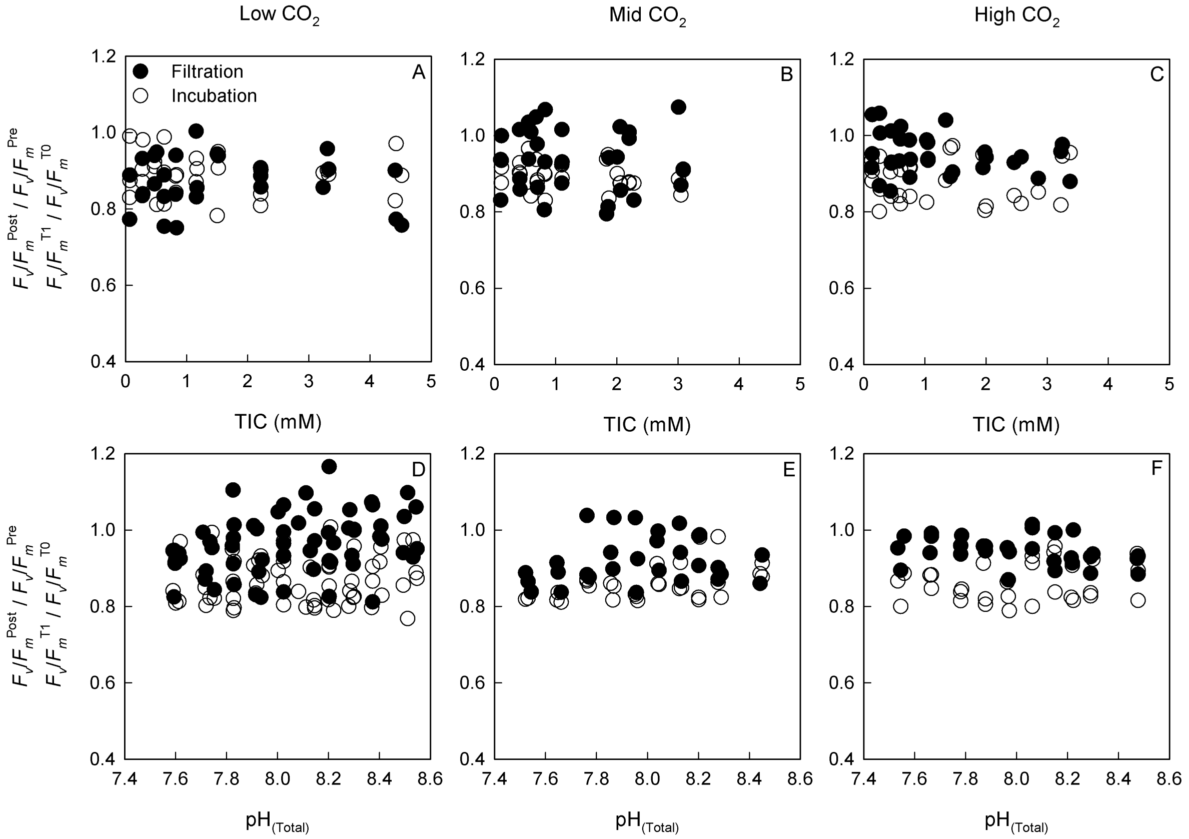

Fig. S1. A post-filtration/re-suspension measure of the dark adapted (~ 20 minutes) photosynthetic efficiency of PSII (F_v/F_m) was made on 2 mL aliquots from each treatment along the TIC (A-C) or pH (D-F) gradient and compared against a pre-filtrated measure of F_v/F_m. The 2 mL samples were kept in the same incubator as the ^{14}C tubes and maintained at identical conditions (i.e. 400 µmol photons m^{-2} s^{-1} and 26 °C ± 0.7 °C). The F_v/F_m was re-measured at the point of ^{14}C-termination (i.e. T1, post incubation) and compared against the post-filtration F_v/F_m (i.e. T0, pre ^{14}C-incubation). Photosynthetic efficiencies were measured using an FRRfII FastAct Fluorometer System (Chelsea Technologies Group Ltd, UK).

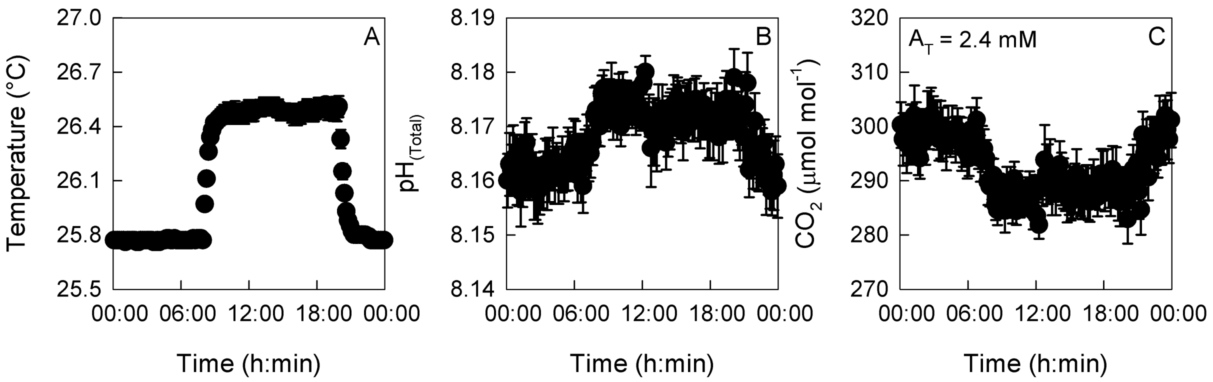

Fig. S2. The inorganic carbon chemistry (Ci) of *T. erythraeum* IMS101 cultures, measured over a diel period. The temperature (°C) and $pH_{(Total)}$ of a mid-CO_2 (~ 380 µmol mol^{-1}) culture was measured with a datalogger (Sper Scientific 840038, Arizona USA) at 10-minute intervals over a 10-day period (mean ± S.E.). The CO_2 drift was calculated via *CO2SYS* using the measured pH and an assumed alkalinity (A_T) concentration of 2.4 mM.

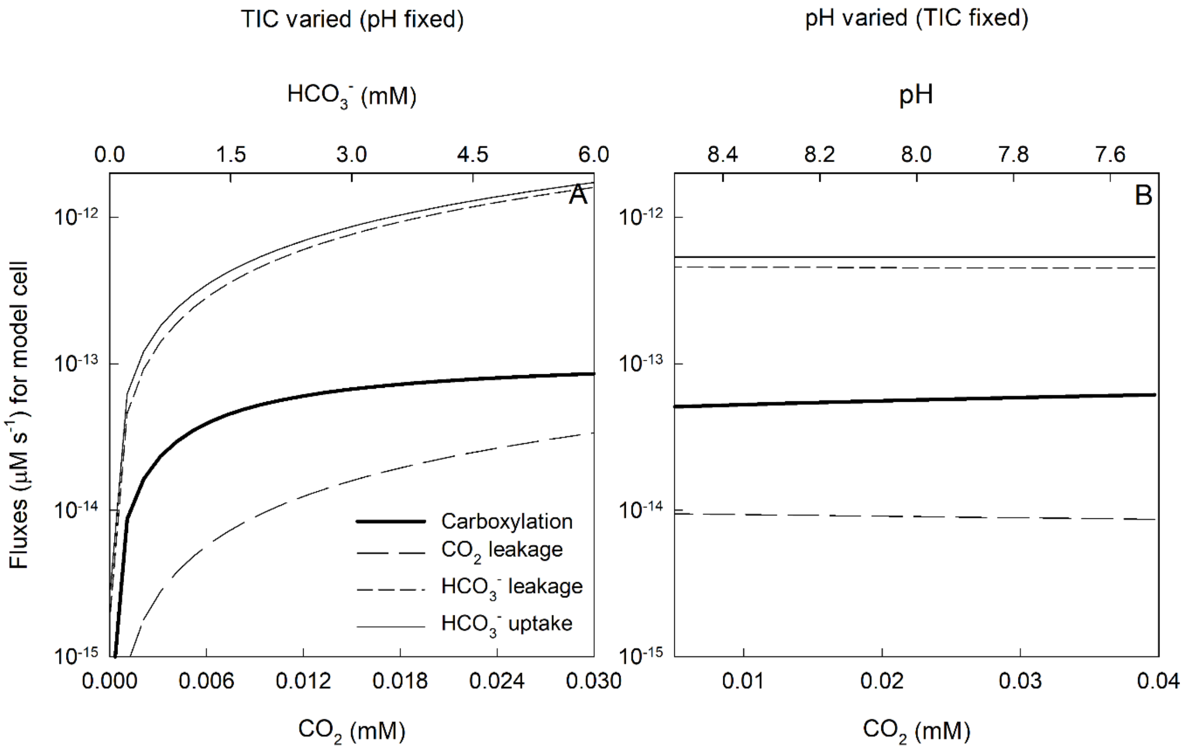

Fig. S3. The calculated rates of carboxylation (bold solid line), CO_2 leakage (long-dashed line), HCO_3^- uptake (solid line) and HCO_3^- leakage (short-dashed line) for a *Trichodesmium* cell, as a function of the external CO_2 (mM); where TIC (i.e. HCO_3-) was varied at a fixed pH = 8.15 (A) and pH was varied at a fixed TIC (HCO_3^- = 1.9 mM) (B).

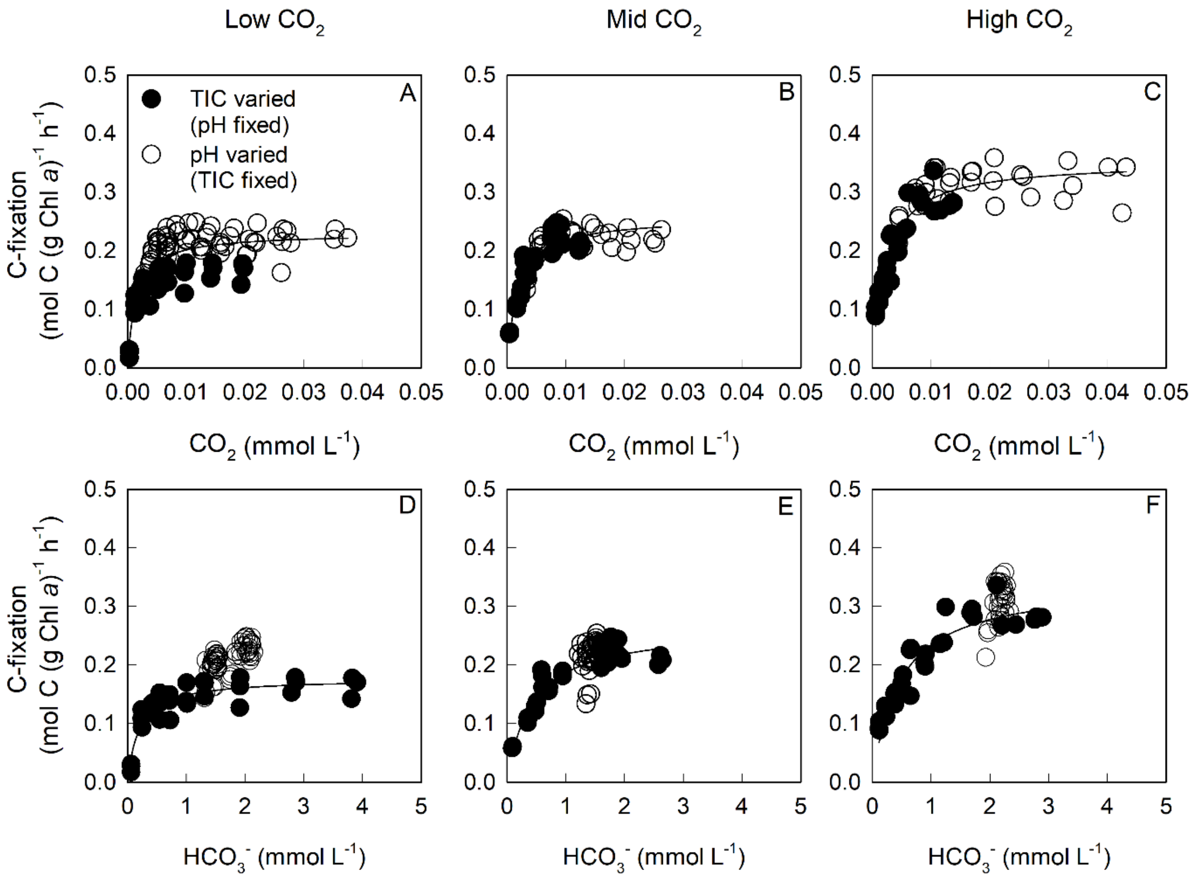

Fig. S4. The combined Chl *a*-specific P-CO_2 and P-HCO_3^- response curves for *T. erythraeum* IMS101, where the two methods of manipulating the inorganic carbon chemistry (Ci) are plot together. Cultures were acclimated to three CO_2 concentrations (Low = 180 µmol mol^{-1}, Mid = 380 µmol mol^{-1} and High = 720 µmol mol^{-1}), saturating light intensity (400 µmol photons m^{-2} s^{-1}) and optimal temperature (26 °C). The CO_2 response curves were generated by manipulating the inorganic carbon chemistry (Ci) using two methods; 1), where TIC and HCO_3^- was varied at a fixed pH (~ 8.15) to achieve a CO_2 gradient (~ 0 – 0.02 mmol L^{-1}) (A-C) and 2), where pH varied (~ 7.52 – 8.54) at a fixed TIC and HCO_3^- to achieve a CO_2 gradient (~ 0 – 0.04 mmol L^{-1}) (D-F). For the CO_2 response, curve fitting was performed using all replicates from both the TIC and pH gradients. For the HCO_3^- response, curve fitting was performed using data from the TIC gradient only. Carbon fixation rates are normalised to a Chl *a* (mol C (g Chl *a*)$^{-1}$ h^{-1}) basis.

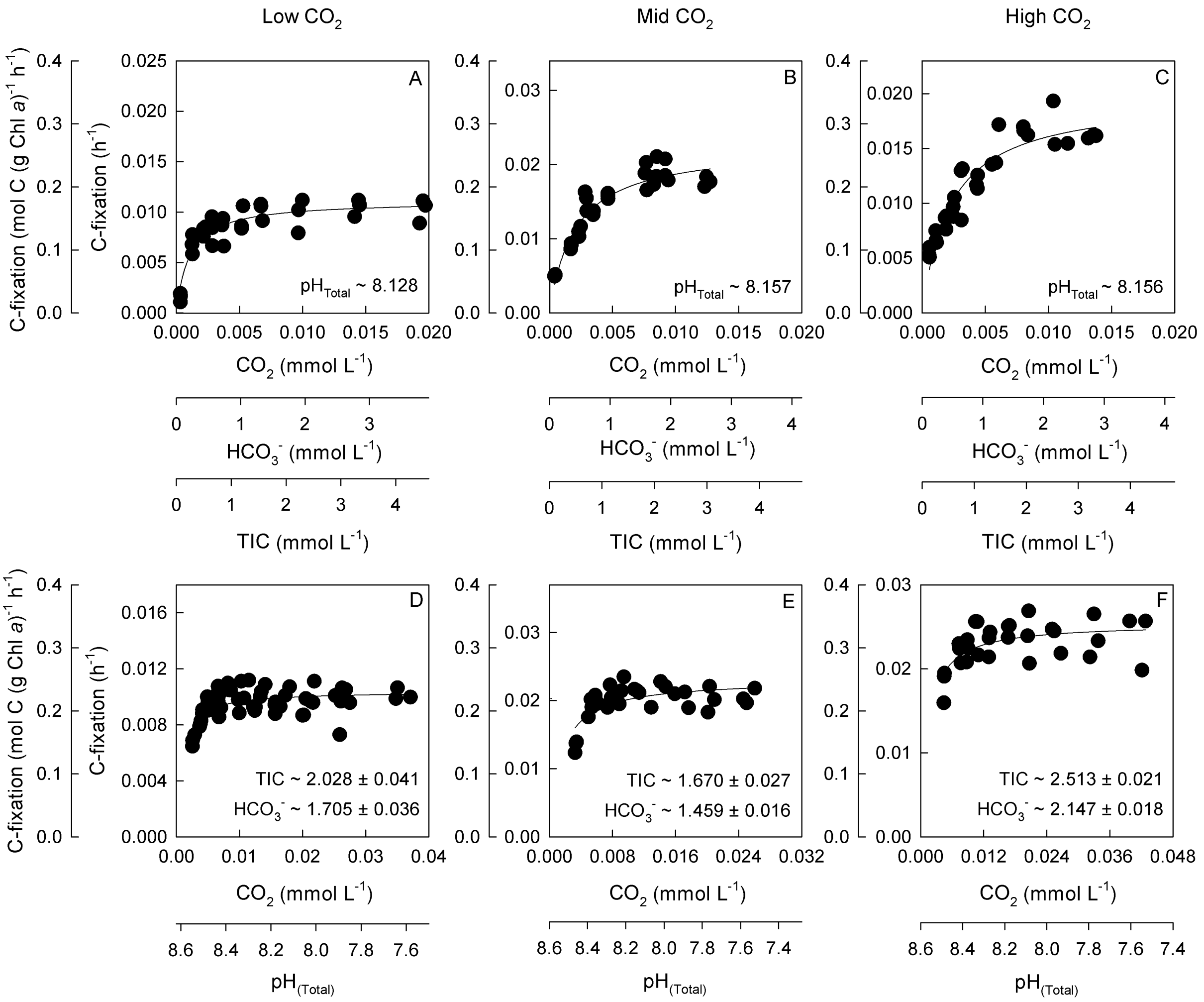

Fig. S5. The combined P-CO_2 response curves (n = 3) for *T. erythraeum* IMS101, using two methods for manipulating the inorganic carbon chemistry (Ci), where TIC and HCO_3^- was varied at a fixed pH (~ 8.15) to achieve a CO_2 gradient (~ 0 – 0.02 mmol L^{-1}) (A-C) and 2), where pH was varied (~ 7.52 – 8.54) at a fixed TIC and HCO_3^- to achieve a CO_2 gradient (~ 0 – 0.04 mmol) (D-F). Cultures were acclimated to three CO_2 concentrations (Low = 180 µmol mol^{-1}, Mid = 380 µmol mol^{-1} and High = 720 µmol mol^{-1}), saturating light intensity (400 µmol photons m^{-2} s^{-1}) and optimal temperature (26 °C). Carbon fixation rates are normalised to a chlorophyll *a* (mol C (g Chl *a*)$^{-1}$ h^{-1}) and carbon (h^{-1}) basis.

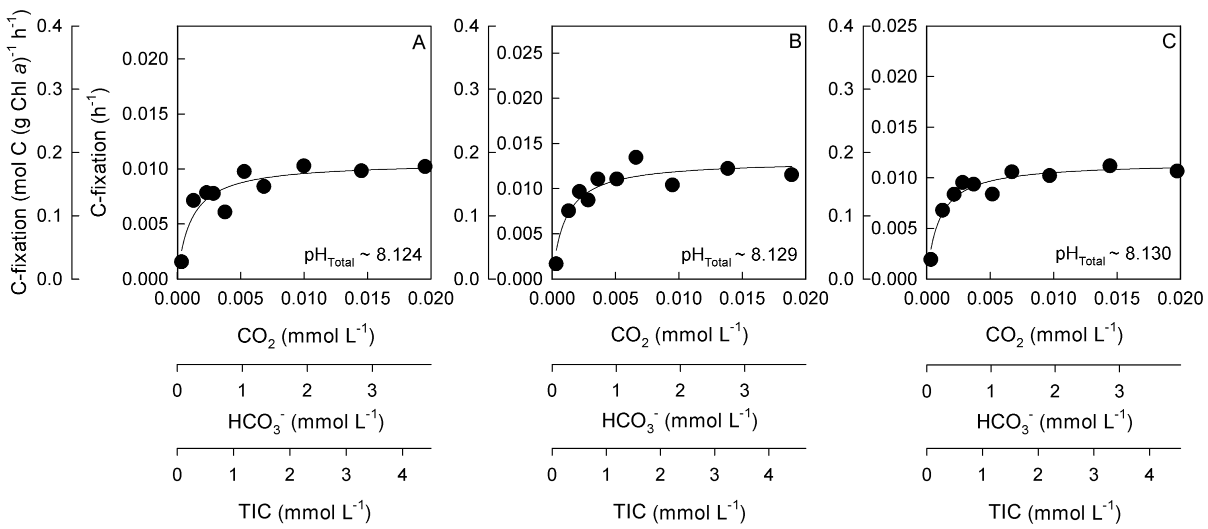

Fig. S6. P-CO_2 response curves for *T. erythraeum* IMS101 when acclimated to low-CO_2, where the TIC and HCO_3^- was varied at a fixed pH (~ 8.13) to achieve a CO_2 gradient (~ 0 – 0.02 mmol L^{-1}). The three-biological replicate (A-C) cultures were acclimated to a low-CO_2 (~ 180 µmol mol^{-1}) concentration, saturating light intensity (400 µmol photons m^{-2} s^{-1}) and optimal temperature (26 °C). Carbon fixation rates are normalised to a chlorophyll *a* (mol C (g Chl *a*)$^{-1}$ h^{-1}) and carbon (h^{-1}) basis.

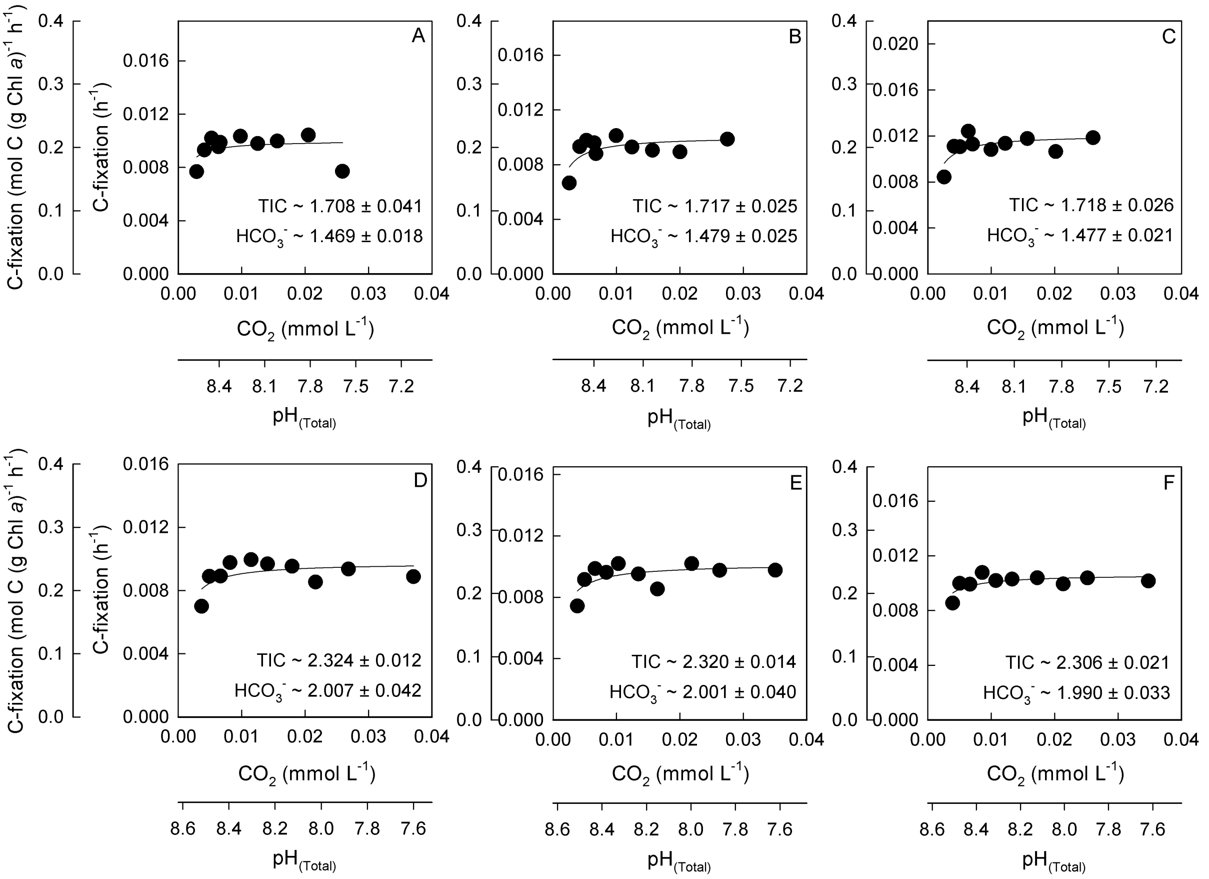

Fig. S7. The P-CO_2 response curves for *T. erythraeum* IMS101 when acclimated to low-CO_2, where the pH was varied (~ 7.59 – 8.55) at a fixed TIC and HCO_3^- to achieve a CO_2 gradient (~ 0 – 0.04 mmol L^{-1}). The six-biological replicate (A-F) cultures were acclimated to a low-CO_2 (~ 180 µmol mol^{-1}) concentration, saturating light intensity (400 µmol photons m^{-2} s^{-1}) and optimal temperature (26 °C). Carbon fixation rates are normalised to a chlorophyll *a* (mol C (g Chl *a*)$^{-1}$ h^{-1}) and carbon (h^{-1}) basis.

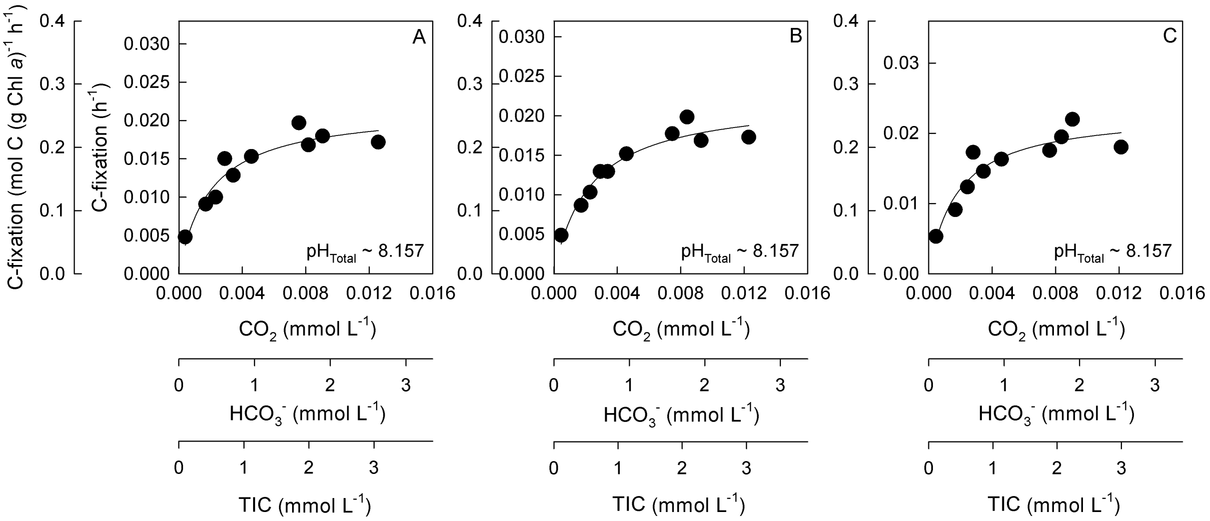

Fig. S8. The P-CO_2 response curves for *T. erythraeum* IMS101 when acclimated to mid-CO_2, where the TIC and HCO_3^- was varied at a fixed pH (~ 8.16) to achieve a CO_2 gradient (~ 0 – 0.013 mmol L^{-1}). The three-biological replicate (A-C) cultures were acclimated to a mid-CO_2 (~ 380 µmol mol^{-1}) concentration, saturating light intensity (400 µmol photons m^{-2} s^{-1}) and optimal temperature (26 °C). Carbon fixation rates are normalised to a chlorophyll *a* (mol C (g Chl *a*)$^{-1}$ h^{-1}) and carbon (h^{-1}) basis.

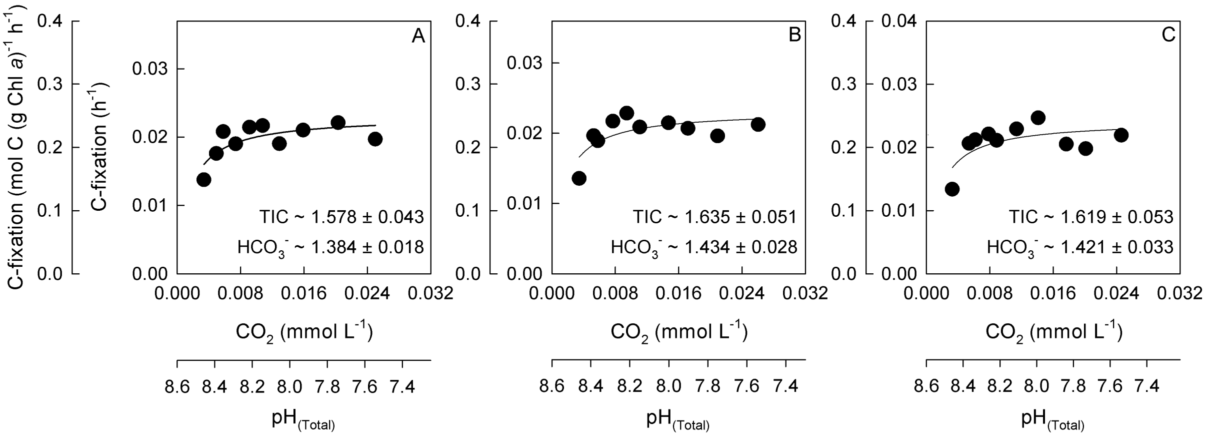

Fig. S9. The P-CO_2 response curves for *T. erythraeum* IMS101 when acclimated to mid-CO_2, where the pH was varied (~ 7.52 – 8.45) at a fixed TIC and HCO_3^- to achieve a CO_2 gradient (~ 0 – 0.04 mmol L^{-1}). The three-biological replicate (A-C) cultures were acclimated to a mid-CO_2 (~ 380 µmol mol^{-1}) concentration, saturating light intensity (400 µmol photons m^{-2} s^{-1}) and optimal temperature (26 °C). Carbon fixation rates are normalised to a chlorophyll *a* (mol C (g Chl *a*)$^{-1}$ h^{-1}) and carbon (h^{-1}) basis.

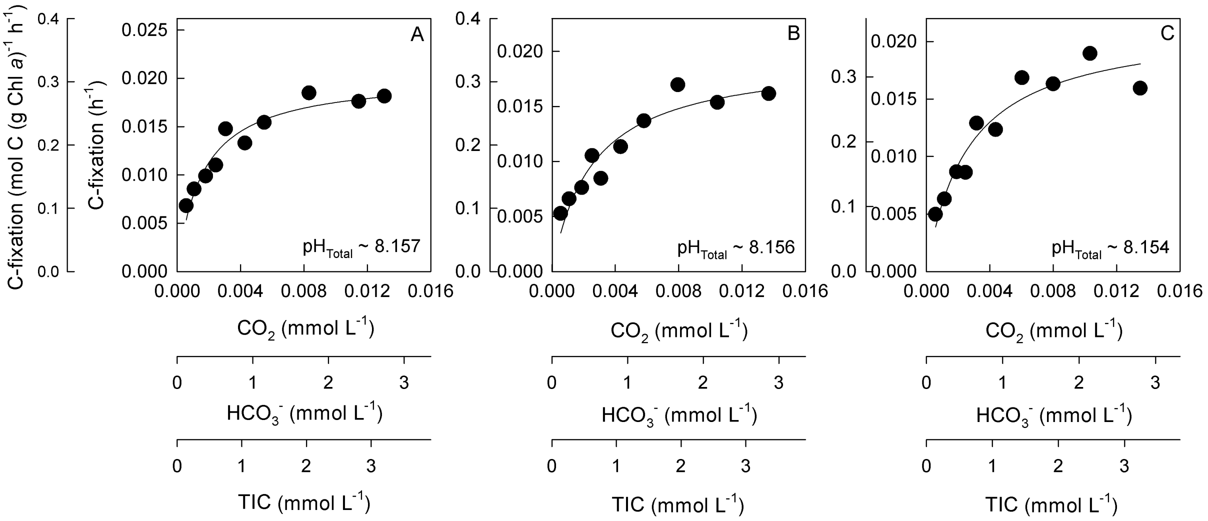

Fig. S10. The P-CO_2 response curves for *T. erythraeum* IMS101 when acclimated to high-CO_2, where the TIC and HCO_3^- was varied at a fixed pH (~ 8.16) to achieve a CO_2 gradient (~ 0 – 0.014 mmol L^{-1}). The three-biological replicate (A-C) cultures were acclimated to a high-CO_2 (~ 720 µmol mol^{-1}) concentration, saturating light intensity (400 µmol photons m^{-2} s^{-1}) and optimal temperature (26 °C). Carbon fixation rates are normalised to a chlorophyll *a* (mol C (g Chl *a*)$^{-1}$ h^{-1}) and carbon (h^{-1}) basis.

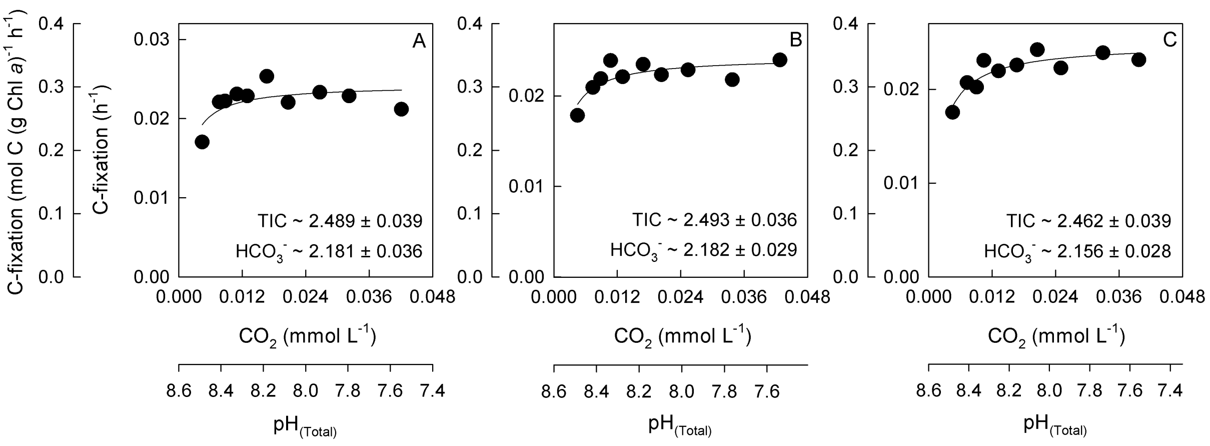

Fig. S11. The P-CO_2 response curves for *T. erythrarum* IMS101 when acclimated to high-CO_2, where the pH was varied (~ 7.53 – 8.48) at a fixed TIC and HCO_3^- to achieve a CO_2 gradient (~ 0 – 0.05 mmol L^{-1}). The three-biological replicate (A-C) cultures were acclimated to a high-CO_2 (~ 720 µmol mol^{-1}) concentration, saturating light intensity (400 µmol photons m^{-2} s^{-1}) and optimal temperature (26 °C). Carbon fixation rates are normalised to a chlorophyll *a* (mol C (g Chl *a*)$^{-1}$ h^{-1}) and carbon (h^{-1}) basis.

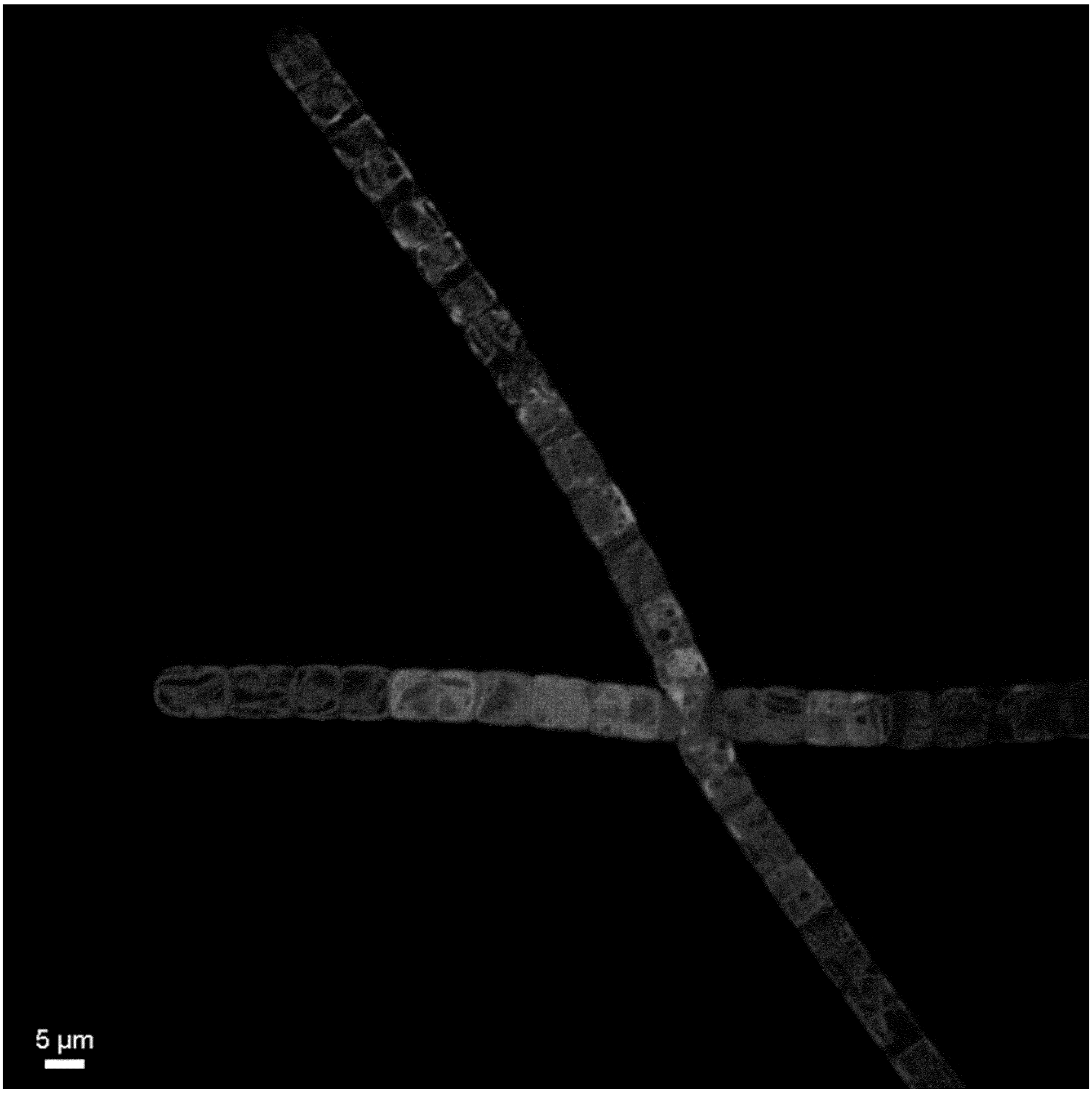

Fig. S12. A bioimage of two *T. erythraeum* IMS101 filaments cultured at mid-CO_2 (~ 380 µatm), saturating light intensity (400 µmol photons m^{-2} s^{-1}) and optimal temperature (26 °C).
